# Higher *RUNX1* expression levels are associated with worse overall and leukaemia‐free survival in myelodysplastic syndrome patients

**DOI:** 10.1002/jha2.547

**Published:** 2022-08-19

**Authors:** Yu‐Hung Wang, Chi‐Yuan Yao, Chien‐Chin Lin, Chi‐Ling Chen, Chia‐Lang Hsu, Cheng‐Hong Tsai, Hsin‐An Hou, Wen‐Chien Chou, Hwei‐Fang Tien

**Affiliations:** ^1^ Division of Hematology Department of Internal Medicine National Taiwan University Hospital Taipei Taiwan; ^2^ Stem Cell and Leukaemia Proteomics Laboratory University of Manchester Manchester UK; ^3^ Graduate Institute of Clinical Medicine College of Medicine National Taiwan University Taipei Taiwan; ^4^ Department of Laboratory Medicine National Taiwan University Hospital Taipei Taiwan; ^5^ Department of Medical Research National Taiwan University Hospital Taipei Taiwan

**Keywords:** leukaemic stem cells signature, myelodysplastic syndrome, prognostication, RUNX1 expression, survival

## Abstract

*RUNX1* mutations are frequently detected in various myeloid neoplasms and implicate unfavourable clinical outcomes in patients with myelodysplastic syndrome (MDS) and acute myeloid leukaemia (AML). On the other hand, high expression of *RUNX1* is also correlated with poor prognosis in AML patients. However, the clinical relevancy of *RUNX1* expression in MDS patients remains elusive. This study aimed to investigate the prognostic and biologic impacts of *RUNX1* expression in MDS patients. We recruited 341 MDS patients who had sufficient bone marrow samples for next‐generation sequencing. Higher *RUNX1* expression occurred more frequently in the patients with Revised International Prognostic Scoring System (IPSS‐R) higher‐risk MDS than the lower‐risk group. It was closely associated with poor‐risk cytogenetics and mutations in *ASXL1*, *NPM1*, *RUNX1*, *SRSF2*, *STAG2*, *TET2* and *TP53*. Furthermore, patients with higher *RUNX1* expression had significantly shorter leukaemia‐free survival (LFS) and overall survival (OS) than those with lower expression. Subgroups analysis revealed that higher‐*RUNX1* group consistently had shorter LFS and OS than the lower‐*RUNX1* group, no matter *RUNX1* was mutated or not. The same findings were observed in IPSS‐R subgroups. In multivariable analysis, higher *RUNX1* expression appeared as an independent adverse risk factor for survival. The prognostic significance of *RUNX1* expression was validated in two external public cohorts, GSE 114922 and GSE15061. In summary, we present the characteristics and prognosis of MDS patients with various *RUNX1* expressions and propose that *RUNX1* expression complement *RUNX1* mutation in MDS prognostication, wherein patients with wild *RUNX1* but high expression may need more proactive treatment.

## 1 INTRODUCTION

Myelodysplastic syndromes (MDSs) represent a heterogeneous group of malignant haematopoietic stem cell (HSC) disorders, with cardinal features of ineffective haematopoiesis, dysplasia of haematopoietic cells, genetic alterations and an inherent propensity of transformation to acute myeloid leukaemia (AML) [1]. The clinical and molecular heterogeneities make these diseases arduous to model and study, highlighting the importance of personalized management [2]. The International Prognostic Scoring System (IPSS) and the revised IPSS (IPSS‐R) have been broadly utilized to risk‐stratify MDS patients and guide treatments [3, 4]. Nonetheless, the prognosis of patients may vary considerably, even within the same risk groups. Therefore, it is crucial to identify novel prognostic biomarkers for better risk classification of patients with MDS.

Besides cytogenetical abnormalities, genetic mutations also correlate with disease phenotypes and clinical outcomes of MDS [5, 6]. For example, mutations in *ASXL1*, *ETV6*, *EZH2*, *RUNX1* and *TP53* identify patients with poor prognoses within each of the IPSS‐R lower risk categories [5, 7]. In addition, aberrant expression of microRNA, long non‐coding RNA, and *BAALC*, *MN1*, and *WT1* genes, as well as genes associated with leukaemia stem cell (LSC) character in the bone marrow (BM), predict poorer prognosis [8, 9, 10, 11, 12, 13]. Among the putative mutational targets, *RUNX1* is imperative for establishing definitive haematopoiesis and is one of the most commonly mutated genes [14]. MDS patients with mutated *RUNX1* had inferior leukaemia‐free survival (LFS) and overall survival (OS) than those without *RUNX1* mutations [15, 16, 17, 18].

Interestingly, growing evidence showed that normal RUNX1 also played a critical role during leukemogenesis. Leukemic cells of core‐binding factor AML and certain types of leukaemia with MLL rearrangements require normal *RUNX1* to survive [14]. More recently, Wesely et al. delicately demonstrated that the *RUNX1* transcription factor is essential for maintaining LSC across various genetic subgroups in AML, implicating RUNX as a potential therapeutic target [19]. Moreover, high expression of *RUNX1* has been shown to be intimately associated with poor prognosis in cytogenetically normal AML (CN‐AML) patients [20]. Meanwhile, although MDS is also considered as LSC‐derived myeloid malignancy, the clinical relevancy of *RUNX1* expression in MDS patients remains obscure. Thus, this study aimed to investigate the prognostic and biologic impacts of *RUNX1* expression in MDS patients.

## 2 METHODS

### 2.1 Patients

We recruited 341 patients with primary MDS diagnosed at the National Taiwan University Hospital (NTUH) from 1997 to 2019 who had adequate cryopreserved BM samples for DNA and RNA sequencing at diagnosis. The diagnosis was based on the 2016 World Health Organization classification [21]. For external validation, we collected publicly annotated RNA‐sequencing data from GSE 114922 [22], where gene expression of BM CD34+ haematopoietic stem and progenitor cells and survival data of 77 MDS patients were available; microarray data from GSE 145733 [23], where gene expression profiles were examined in CD34+ BM cells from 54 patients with MDS and 14 patients with AML with myelodysplasia‐related changes (AML‐MRC); and GSE15061, where gene expression and survival data of 110 MDS patients were available [24]. The Research Ethics Committee of the NTUH approved this study (approval number: 201709072RINC), and written informed consent was provided according to the Declaration of Helsinki.

### 2.2 Cytogenetic study and molecular mutation analysis by targeted next‐generation sequencing (NGS)

Cytogenetic analyses were performed as previously described and interpreted according to the International System for Human Cytogenetic Nomenclature [25, 26]. We employed the TruSight myeloid sequencing panel and the HiSeq platform to analyze gene alterations and mutant allele burden of 54 myeloid‐neoplasm relevant genes (Table S1) as previously described [27] on BM samples of 333 MDS patients. The library preparation and sequencing were following the manufacturer's instructions. The Catalogue Of Somatic Mutations In Cancer database version 86 [28], ClinVar [29], dbSNP database version 151 [30], PolyPhen‐2 (Polymorphism Phenotyping v2) [31], and SIFT [32] were used to evaluate the result of each variant.

### 2.3 Library preparation and RNA sequencing

We prepared the sequencing library with purified RNA as previously described [33], using the TruSeq Stranded mRNA Library Prep Kit (Illumina, San Diego, CA, USA) and following the manufacturer's recommendations. The detailed methods are described in Supplementary Method 1.

### 2.4 Bioinformatic analysis and statistical analysis

The normalized signals for RNA sequencing data were analyzed using the pre‐ranked gene set enrichment analysis (GSEA) implemented in the R package clusterProfiler, with gene sets downloaded from the Molecular Signature Database (MSigDB) database. Other statistical methods are detailed in Supplementary Method 2.

## 3 RESULTS

### 3.1 Patient characteristics

The patient characteristics are summarized in Table 1. The median age of the 341 MDS patients was 68.3 years. Among the 332 patients who had cytogenetic data at diagnosis, 4.5%, 24.1%, 23.5%, 24.4% and 23.5% had IPSS‐R very‐low risk, low risk, intermediate risk, high risk and very‐high risk MDS, respectively. In total, 48.1% of patients received supportive care only, and 177 received active treatment, including hypomethylating agents, low‐dose cytarabine and AML‐directed intensive chemotherapy at the time of leukemic transformation or as a bridge to allogeneic hematopoeitic stem cell transplantation (allo‐HSCT). Overall, 59 (17.3%) patients received allo‐HSCT. During a median follow‐up duration of 32.9 months (range, 0.2–165.4 months), 117 patients (34%) progressed to AML, and 158 patients succumbed to the disease.

**TABLE 1:** Comparison of clinical and laboratory features between patients with lower and higher RUNX1 expression

**Clinical characters**	**Total (*n* = 341)**	**Lower *RUNX1* (*n* = 171)**	**Higher *RUNX1* (*n* = 170)**	***p‐*Value**
**Sex**				0.737
**Female**	125 (36.7)	61 (35.7)	64 (37.6)	
**Male**	216 (63.3)	110 (64.3)	106 (62.4)	
**Age** ^a^	68.3 (18.0–94.2)	65.4 (20.0–94.2)	69.3 (18.0–93.1)	0.093
**Laboratory data** *				
**WBC, X 10^9^ /L**	3.68 (0.31–54.4)	3.85 (0.49–24.3)	3.62 (0.31–54.4)	0.357
**ANC, X 10^9^ /L**	1.70 (0.01–3.71)	1.95 (0.03–19.4)	1.61 (0.01–3.71)	0.192
**Hb, g/dl**	8.4 (4–17)	8.3 (4–17)	8.4 (4–15)	0.551
**Platelet, X 10^9^ /L**	83 (1–721)	103.5 (3–669)	72 (1‐721)	0.035
**PB blast (%)**	0 (0–18)	0 (0–14)	1 (0–18)	<0.001
**BM blast (%)**	6 (0–19)	2.7 (0–18.6)	9.2 (0–19.0)	<0.001
**2016 WHO classification**				<0.001
**MDS‐SLD**	35 (10.3)	30 (17.5)	5 (2.9)	
**MDS‐MLD**	50 (14.7)	33 (19.3)	17 (10.0)	
**MDS‐RS**	26 (7.6)	19 (11.1)	7 (4.1)	
**MDS‐RS‐MLD**	24 (7.0)	19 (11.1)	5 (2.9)	
**MDS‐U**	5 (1.5)	3 (1.8)	2 (1.2)	
**MDS‐EB1**	84 (24.6)	32 (18.7)	52 (30.6)	
**MDS‐EB2**	117 (34.3)	35 (20.5)	82 (48.2)	
**IPSS‐R** ^†^, ^‡^				<0.001
**Very low**	15 (4.5)	12 (7.3)	3 (1.8)	
**Low**	80 (24.1)	57 (34.5)	23 (13.8)	
**Intermediate**	78 (23.5)	46 (27.9)	32 (19.2)	
**High**	81 (24.4)	29 (17.6)	52 (31.1)	
**Very high**	78 (23.5)	21 (12.7)	57 (34.1)	
**Treatment**				
**Supportive care**	164 (48.1)	103 (60.2)	61 (35.9)	<0.001
**Active treatment** ^§^	177 (51.9)	68 (39.8)	109 (64.1)	
**Hypomethylation agents**	134 (39.3)	49 (28.7)	85 (50.0)	<0.001
**LDAraC** ^¶^	30 (8.8)	8 (4.7)	22 (12.9)	0.007
**Intensive chemotherapy**	33 (9.7)	8 (4.7)	25 (14.7)	0.002
**HSCT**	59 (17.3)	28 (16.4)	31 (18.2)	0.670

*Note*: Statistically significant if *p*‐value < 0.05.

Abbreviations: ANC, absolute neutrophil count; BM, bone marrow; Hb, hemoglobin; HSCT, allogeneic hematopoietic stem cell transplantation.; IPSS‐R, revised international prognosis scoring system; LDAraC: low‐dose cytarabine; MDS‐EB, MDS with excess blasts; MDS‐MLD, MDS with multilineage dysplasia; MDS‐RS, MDS with ring sideroblasts; MDS‐RS‐MLD, MDS with ring sideroblasts and multilineage dysplasia; MDS‐RS‐SLD, MDS with ring sideroblasts and single lineage dysplasia; MDS‐SLD, MDS with single lineage dysplasia; MDS‐U, MDS, unclassifiable; PB, peripheral blood.

* Median (range).

^†^ 332 patients, including 165 with lower *RUNX1* expression and 167 with *higher* RUNX1 expression, had chromosome data at diagnosis.

^‡^ IPSS‐R: Very low, ≦1.5; Low, > 1.5‐3; intermediate, > 3‐4.5; High, > 4.5‐6; and Very high, > 6.

^§^ Active treatment: HMA, LDAraC, high intensity chemotherapy, and HSCT. Some patients received more than one treatment modality: 14 received HMA and LDAraC, 14 received HMA and high intensity chemotherapy, three received LDAraC and high intensity chemotherapy; four received high intensity chemotherapy and HSCT; 37 received HMA and HSCT; three received HMA, high intensity chemotherapy and HSCT; one received HMA, LDAraC and HSCT; and 14 received HSCT without bridging therapy.

^¶^ low‐dose cytarabine: at 20 mg once or twice daily for 10 consecutive days every 4–6 weeks.

### 3.2 Comparison of clinical characteristics and genetic alterations between patients with higher and lower *RUNX1* expression

**FIGURE 1:**
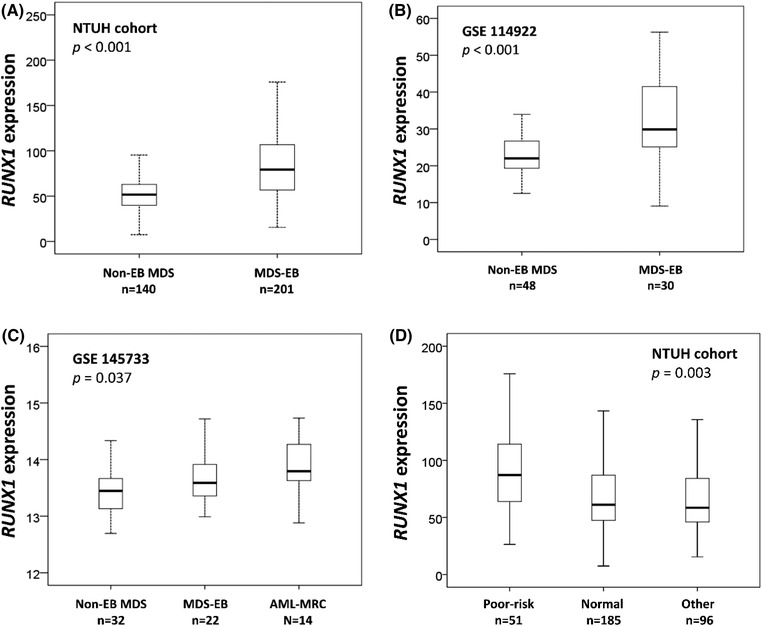
Box plot depicting *RUNX1* expression in patients with myelodysplastic syndromes (MDS) with or without excess blasts (excess blasts [EBs] and non‐EB, respectively) from the National Taiwan University Hospital (NTUH), GSE 114922 and GSE 145733 cohorts, and in patients with acute myeloid leukaemia with myelodysplasia‐related changes (AML‐MRC) from the GSE 145733 cohort; and patients with various karyotypes in the NTUH cohort. (A) Patients with MDS‐EB had significantly higher *RUNX1* expression than those with non‐EB MDS in the NTUH cohort. (B) Patients with MDS‐EB had significantly higher *RUNX1* expression than those with non‐EB MDS in the GSE 114922 cohort. (C) Patients with AML‐MRC had significantly higher *RUNX1* expression than those with MDS‐EB or non‐EB MDS in the GSE 145733 cohort. (D) Patients with poor‐risk karyotypes had significantly higher *RUNX1* expression than those with normal or other karyotypes in the NTUH cohort.

Histograms representing the distribution of *RUNX1* expression were plotted in Figure S1A. We first explored the expression of *RUNX1* in various IPSS‐R subgroups and found that patients with higher‐risk IPSS‐R had higher expression of *RUNX1*. Additionally, Pearson's correlation revealed that *RUNX1* expression significantly correlated with IPSS‐R subgroups (*r* = 0.41, *p* < 0.001, Figure S1B). Specifically, patients with MDS with excess blasts (MDS‐EBs) had substantially higher *RUNX1* expression than those with non‐EB MDS (*p* < 0.001, Figure 1A). The same was true in the GSE 114922 cohort, wherein gene expression of BM CD34+ cells was available (*p* < 0.001, Figure 1B). More intriguingly, in the GSE 145733 cohort, in which gene expression of CD34+ cells was analysed, *RUNX1* expression was significantly correlated with blast counts (*r* = 0.355, *p* = 0.003) while patients with AML‐MRC had higher *RUNX1* expression than those with either MDS‐EB or non‐EB MDS (*p* = 0.037, Figure 1C), indicating that *RUNX1* might play a role during the acute transformation of MDS. Meanwhile, patients with poor‐risk karyotype had higher *RUNX1* expression than those with normal karyotypes and others in the NTUH cohort (*p* = 0.003, Figure 1D).

We next examined the difference in *RUNX1* expression between patients with wild and mutated *RUNX1*. Patients with *RUNX1* mutations had higher *RUNX1* expression than unmutated patients. The expression was remarkably higher in those carrying C‐terminal mutations than others (median, unmutated vs. N‐terminal mutated vs. C‐terminal mutated: 59.6 vs. 81.2 vs. 95.4, *p* < 0.001), as illustrated in Figure S1C. The mutation details and expression levels of *RUNX1* in patients with mutant *RUNX1* are displayed in Table S2.

The 341 MDS patients were subsequently divided into the higher‐ and lower‐ *RUNX1* expression (higher‐ and lower‐ *RUNX1*) groups with the median value (62.49 TPM) of the *RUNX1* expression as the cut‐off level. The comparison of clinical and laboratory features between the two groups is presented in Table 1. The higher‐*RUNX1* group had lower platelet counts (*p* = 0.035), higher blast percentages in the BM (*p* < 0.001) and peripheral blood (*p* < 0.001) at diagnosis compared to the lower‐*RUNX1* groups. The higher‐*RUNX1* patients more frequently had MDS‐EB, including MDS‐EB1 and MDS‐EB2, but less MDS with single lineage dysplasia (MDS‐SLD), MDS with multilineage dysplasia (MDS‐MLD), MDS with ring sideroblasts and single lineage dysplasia (MDS‐RS‐SLD), and MDS with ring sideroblasts and multilineage dysplasia (MDS‐RS‐MLD) compared to the lower‐*RUNX1* group (*p* < 0.001). Furthermore, the higher‐*RUNX1* patients had higher frequencies of IPSS‐R high and very‐high risk MDS but lower frequencies of low and very‐low risk MDS (*p* < 0.001). Complex karyotypes were more common in the higher‐*RUNX1* patients than the lower ones (19.8% vs. 6.7%, *p* = 0.001, Table S3). There were also more higher‐*RUNX1* patients harbouring poor or very poor risk karyotypes per IPSS‐R classification (23.4% vs. 7.3%, *p* < 0.001).

Regarding molecular gene alterations, 260 (78.1%) of the 333 patients with available data had at least one mutation in the 54 genes analysed. As listed in Table S4, the most common mutation was *ASXL1* mutation (24.9%), followed by *RUNX1* (16.2%), *SF3B1* (14.7%), *TET2* (13.8%) and *TP53* mutations (12.9%). Higher *RUNX1* expression was closely associated with mutations in *ASXL1*, *NPM1*, *RUNX1*, *SRSF2*, *STAG2*, *TET2*, *TP53* and *ZRSR2* (with respective *p* values of 0.002, 0.003, <0.001, <0.001, 0.049, 0.011, 0.001 and 0.027, Table S4) whereas lower‐*RUNX1* expression, with *SF3B1* mutation (*p* = 0.002).

### 3.3 The effects of *RUNX1* expression on LFS and OS

Parameters including age, sex and those associated with *RUNX1* expression (as shown above) were examined consecutively for their potential of confounding with *RUNX1* expression (Table S5). By threshold of 10% change in the hazard ratio (HR), IPSS‐R, excess of blasts, poor‐risk karyotypes and TP53 mutation were identified as confounders for OS (HR change, 26.4%, 25.1%, 13.0%, and 11.9%, respectively). As the IPSS‐R score is determined by clinical features that include the excess of blast and poor‐risk karyotypes, we therefore, applied IPSS‐R and TP53 in the subsequent tests for the adjustment of HRs where possible (mutation profile was not available in the validation sets).

**FIGURE 2:**
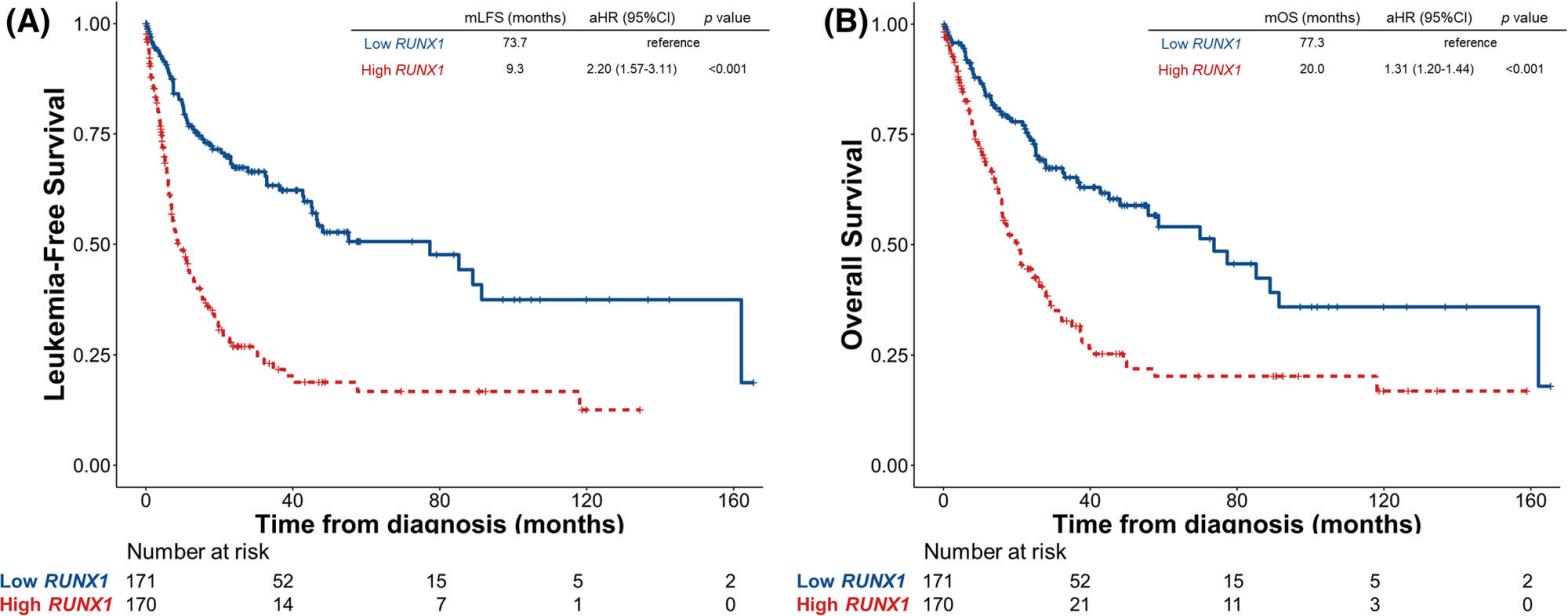
Kaplan–Meier survival curves stratified by *RUNX1* expression in the total cohort. (A) Leukaemia‐free survival (LFS) and (B) overall survival (OS) of the 341 myelodysplastic syndrome (MDS) patients. Patients with higher *RUNX1* expression had inferior LFS and OS than those with lower expression.

**FIGURE 3:**
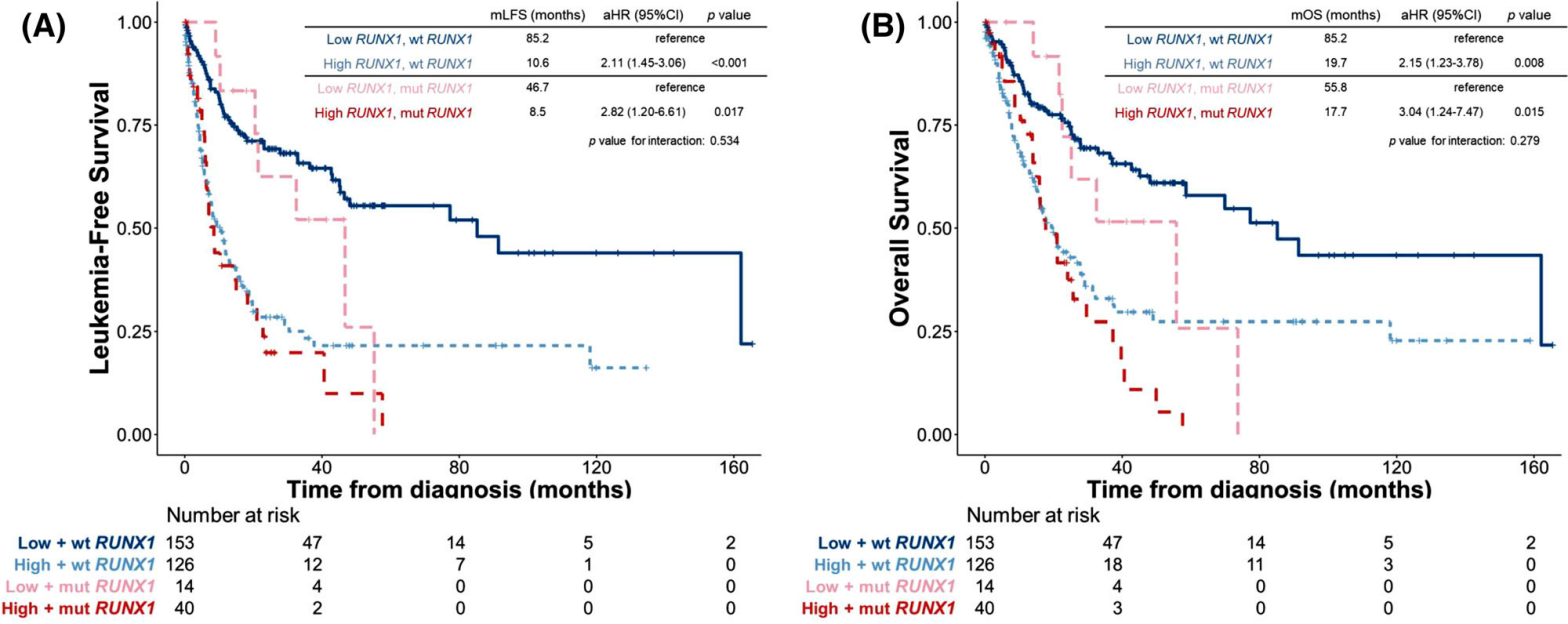
Kaplan–Meier survival curves stratified by *RUNX1* expression and *RUNX1* mutation status in total cohort. (A) Leukaemia‐free survival (LFS) and (B) overall survival (OS) of the 279 myelodysplastic syndrome (MDS) patients with wild *RUNX1* and the 54 MDS patients with mutated *RUNX1*. Patients with higher *RUNX1* expression had significantly inferior LFS and OS despite their *RUNX1* mutation status.

**FIGURE 4:**
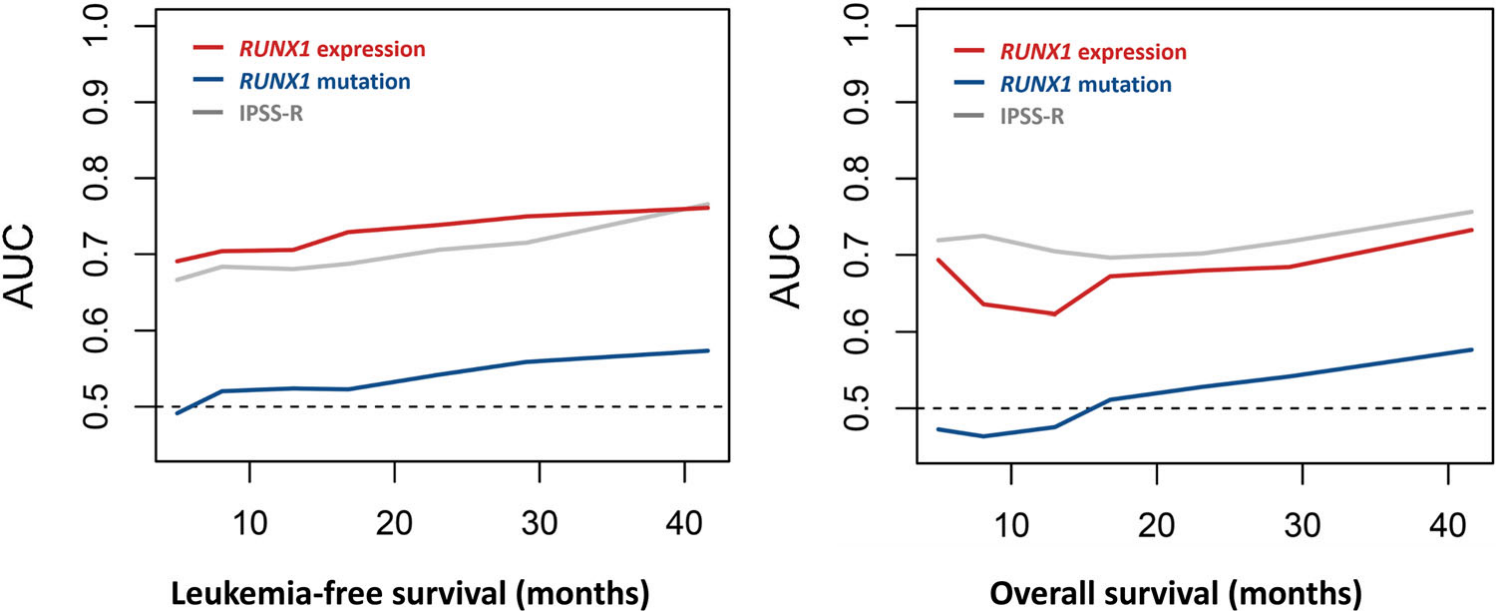
Time‐dependent ROC curve analyses showing that the *RUNX1* expression had better predictive power for leukaemia‐free survival (LFS) and overall survival (OS) than *RUNX1* mutation. ROC curves were estimated by inverse probability of censoring weighting. AUC, area under the curve

**FIGURE 5:**
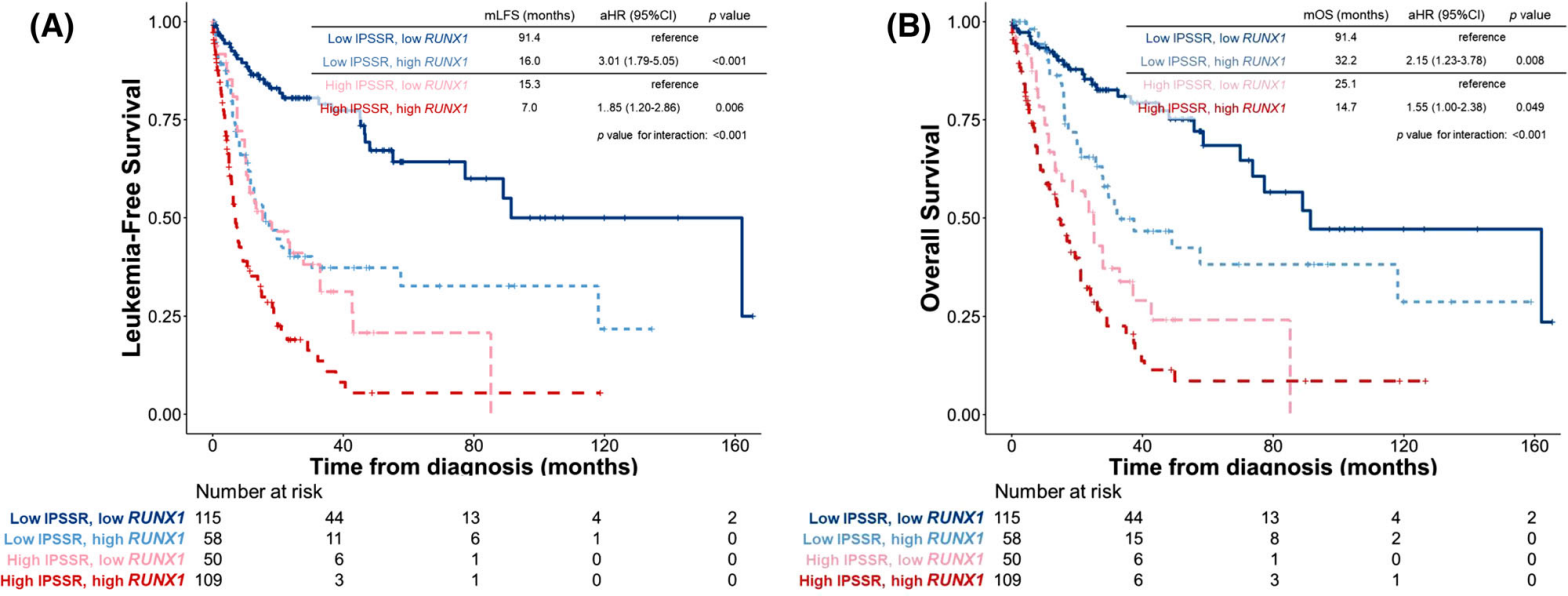
Kaplan–Meier survival curves stratified by *RUNX1* expression and Revised International Prognostic Scoring System (IPSS‐R) leukaemia‐free survival (LFS) (A) and overall survival (OS) (B) of the 332 patients in the training cohort who had cytogenetic data at diagnosis (thus IRSS‐R could be calculated). Patients with higher *RUNX1* expression had significantly inferior LFS and OS despite their IPSS‐R risk. Patients with higher *RUNX1* expression and high IPSS‐R had the worst outcomes while those with lower *RUNX1* expression and low IPSS‐R had the best outcomes.

We first examined the impact of *RUNX1* mutation on survival. Conceivably, patients with mutated *RUNX1* had significantly inferior LFS and OS than those with unmutated *RUNX1* (adjusted HR [aHR], 1.67, 95% confidence interval [95% CI] 1.15–2.43, *p* = 0.022 and aHR 1.68, 95% CI 1.14–2.49, *p* = 0.009, respectively, Figure S2). We next queried the effects of *RUNX1* expression on patients’ survival. Patients with higher *RUNX1* expression had significantly shorter LFS and OS than those with lower expression (aHR 2.20, 95% CI 1.57–3.11, *p* < 0.001; and aHR 1.31, 95% CI 1.20–1.44, *p* < 0.001, respectively, Figure 2A,B). We further incorporated both *RUNX1* mutation statuses and *RUNX1* expression into risk stratification. The higher‐*RUNX1* group consistently had reduced LFS and OS than the lower‐*RUNX1* group despite *RUNX1* mutation statuses (wild *RUNX1*, aHR 2.11, 95% CI 1.45–3.06, *p* < 0.001; and aHR 2.82, 95% CI 1.20–6.61, *p* = 0.017, respectively, Figure 3A,B; and mutated *RUNX1*, aHR 2.82, 95% CI 1.20–6.61, *p* = 0.017; and aHR 3.04, 95% CI 1.24–7.47, *p* = 0.015, respectively, Figure 3C,D). Moreover, time‐dependent receiver operating characteristic (ROC) curves indicated that *RUNX1* expression had better predictive power for LFS and OS than *RUNX1* mutation (Figure 4). Subgroups analyses of IPSS‐R risk groups also revealed that higher *RUNX1* expression conferred inferior LFS and OS in both IPSS‐R lower‐risk (very low, low and intermediate‐risk) patients (aHR 3.01, 95% CI 1.79–5.05, *p* < 0.001; and aHR 2.15, 95% CI 1.23–3.78, *p* = 0.008, respectively, Figure 5A,B) as well as higher‐risk (high and very high risk) patients (aHR 1.85, 95% CI 1.20–2.86, *p* = 0.006; and aHR 1.55, 95% CI 1.002–2.380, *p* = 0.049, respectively, Figure 5C,D). Collectively, *RUNX1* expression could be complementary to *RUNX1* mutation and IPSS‐R to refine the risk stratification of patients with MDS.

The prognostic implications of *RUNX1* expression on LFS and OS were also demonstrated in the subgroups of patients with normal karyotype (*n* = 185, aHR 2.69, 95% CI 1.59–4.56, *p* < 0.001; and aHR 2.26, 95% CI 1.29–3.98, *p* = 0.005, respectively, Figure 3A,B) and patients without unfavourable cytogenetics such as complex karyotypes and monosomy 7 (*n* = 281, aHR 2.34, 95%CI 1.59–3.44, *p* < 0.001; and aHR 1.79, 95% CI 1.19–2.71, *p* = 0.001, respectively, Figure S3C,D). We further analysed the influence of *RUNX1* expression on clinical outcomes of MDS patients receiving different treatment regimens. The higher‐*RUNX1* patients consistently had inferior LFS and OS (Figure S4), no matter they received supportive care (*n* = 164, aHR 2.36, 95% CI 1.28–4.38, *p* = 0.006; and aHR 2.59, 95% CI 1.38–4.89, *p* = 0.003, respectively) or active treatment (*n* = 177, aHR 2.06, 95% CI 1.36–3.11, *p* = 0.001; and aHR 1.62, 95% CI 1.05–2.48, *p* = 0.028, respectively). Among the 134 patients receiving hypomethylation agents, the differences in LFS and OS between the two groups remained significant (aHR 2.06, 95% CI 1.24–3.40, *p* = 0.005; and aHR 1.85, 95% CI 1.11–3.09, *p* = 0.019, respectively, Figure S5). Intriguingly, for the patients receiving allo‐HSCT, the detrimental effect of higher‐*RUNX1* expression seemed to be partly alleviated. Among patients with higher‐*RUNX1* expression, those who did not received allo‐HSCT had significantly worse LFS and OS than those receiving allo‐HSCT (aHR 2.37, 95% CI 1.39–4.04, *p* = 0.002; and aHR 2.71, 95% CI 1.55–4.74, *p* < 0.001, respectively, Figure S6A,B). Additionally, higher‐*RUNX1* patients who underwent allo‐HSCT had a comparable OS to lower‐*RUNX1* patients with or without HSCT (Figure S6B).

In multivariable analysis, we included age, sex and the confounders IPSS‐R and *TP53* mutations in the analysis for LFS and OS. Higher *RUNX1* expression, either divided by a median (Table 2) or regarded as continuous values (Table S6), appeared to be an independent adverse prognostic factor for LFS (aHR 2.114, 95% CI 1.506–2.969, *p* < 0.001 and aHR 1.009, 95% CI 1.005–1.014, *p* < 0.001, respectively) and OS (aHR 1.721, 95% CI 1.211–2.445, *p* = 0.002 and aHR 1.007, 95% CI 1.002–1.012, *p* = 0.004, respectively). By virtue of the association between higher *RUNX1* expression and BM blast fractions and poor‐risk karyotypes, respectively, we hypothesized whether the prognostic value of *RUNX1* expression levels could simply originate from the other two parameters. Hence, we performed multivariable analysis adopting BM blast percentages, poor‐risk karyotypes and *RUNX1* expression as variables. Again, *RUNX1* expression remained an independent prognostic factor of LFS and OS, either by dichotomy (aHR 2.032, 95% CI 1.453–2.841, *p* < 0.001; and aHR 1.591, 95% CI 1.116–2.270, *p* = 0.010, respectively) or continuous variable (aHR 1.008, 95% CI 1.004–1.012, *p* < 0.001; and aHR 1.005, 95% CI 1.000–1.010, *p* = 0.032, respectively).

**TABLE 2:** Multivariable analysis for LFS and OS in the 324 MDS patients who had both cytogenetic data and NGS mutation data at diagnosis

	LFS				OS			
	95% CI				95% CI			
Variable	**aHR**	**Lower**	**Upper**	***p‐*Value**	**aHR**	**Lower**	**Upper**	***p‐Value***
Age*	1.018	1.007	1.03	0.001	1.032	1.019	1.044	<0.001
Sex (reference: female)	1.208	0.872	1.675	0.256	1.419	0.998	2.018	0.051
IPSS‐R^†^	1.302	1.188	1.427	<0.001	1.382	1.256	1.521	<0.001
*TP53*	2.423	1.597	3.677	<0.001	4.682	3.014	7.273	<0.001
Higher *RUNX1* expression^‡^	2.114	1.506	2.969	<0.001	1.721	1.211	2.445	0.002

*Note*: Statistically significant if *p* < 0.05.

Abbreviations: aHR, adjusted hazard ratios; CI, confidence interval, IPSS‐R, Revised International Prognostic Scoring System; LFS, leukaemia‐free survival; MDS, myelodysplastic syndrome; OS, overall survival.

* Age, as a continuous variable analysis.

^†^ IPSS‐R risk groups: Very good, good, intermediate, poor, very poor.

^‡^ High versus low *RUNX1* expression (median as cutoff).

**FIGURE 6:**
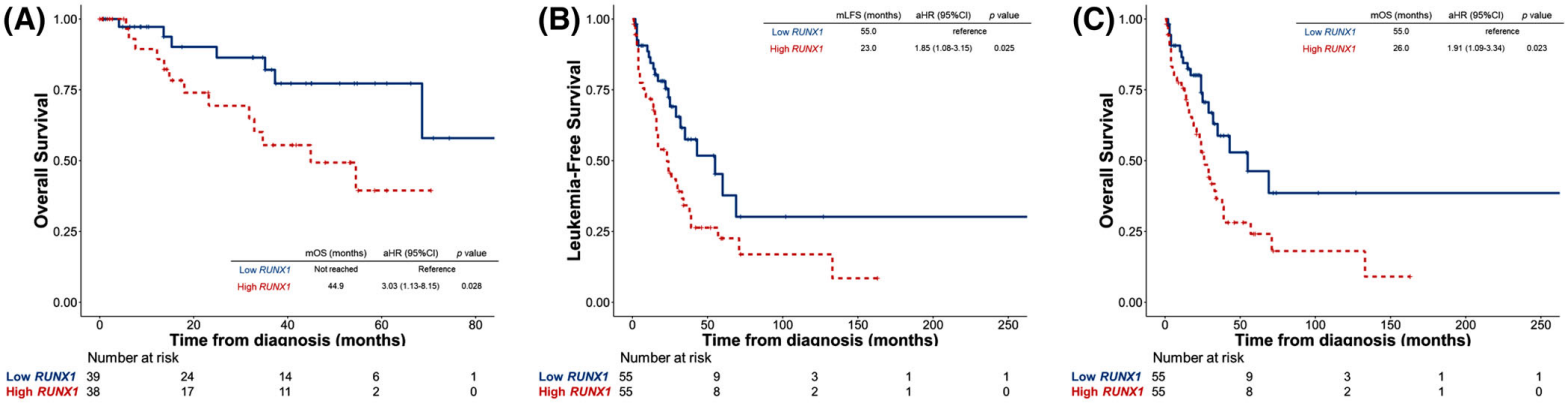
Kaplan–Meier survival curves of myelodysplastic syndrome (MDS) patients stratified by *RUNX1* expression in two independent validation cohorts. (A) Overall survival (OS) of 77 MDS patients in an external validation cohort from GSE 114922; and (B) leukaemia‐free survival (LFS) and (C) OS of 110 MDS patients in an external validation cohort from GSE15061. Patients with higher *RUNX1* expression consistently had inferior clinical outcomes. There was no annotated LFS data in GSE 114922.

The prognostic significance of *RUNX1* expression was further validated in two external public cohorts, GSE 114922 and GSE15061. Patients were divided into higher‐ and lower‐*RUNX1* expression groups with median values as cut‐offs in each dataset (25.1 and 0.54, respectively). The comparisons of clinical and laboratory features between higher‐ and lower‐*RUNX1* patients in the two cohorts are displayed in Tables S8 and S9, respectively. Consistently, patients with higher‐*RUNX1* expression had worse outcomes than those with lower‐*RUNX1* expression in both cohorts (GSE114922, OS, aHR 3.03, 95% CI 1.13–8.15, *p* = 0.028; and GSE15061, LFS, aHR 1.85, 95% CI 1.08–3.15, *p* = 0.025; and OS, aHR 1.91, 95% CI 1.09–3.34, *p* = 0.023, respectively, Figure 6). Sensitivity analysis revealed no significant heterogeneity detected across the training cohort and validation cohorts regarding OS rate at 5 years (*p* = 0.26, *I* ^2^ = 26%).

### 3.4 Biological implication of higher *RUNX1* expression

Following the above observations, we aimed to explore the potential mechanistic insight into how higher *RUNX1* expression affects MDS biology and prognosis. GSEA showed that core enriched HSC/leukaemic stem cells (CE‐HSC/LSC) signature and various annotated HSC‐ or LSC‐relevant signatures were significantly enriched in patients with higher *RUNX1* expression than their lower *RUNX1* expression counter partners (Figure S7), suggesting its role in maintaining and promoting leukaemia growth.

## 4 DISCUSSION

To the most of our knowledge, this is the first study to investigate the prognostic significance of *RUNX1* expression levels in MDS patients. We found that the patients with higher *RUNX1* expression showed distinct clinical and biological characteristics and had shorter LFS and OS. Higher *RUNX1* expression was an independent poor prognostic factor, irrespective of other risk factors in MDS patients. Furthermore, the prognostic implication of *RUNX1* expression remained significant in both IPSS‐R higher‐ and lower‐risk patients as well as *RUNX1*‐mutated and wild‐type groups.

*RUNX1* encodes the DNA binding alpha subunit of the core binding transcription factor, which is a pivotal regulator of definitive haematopoiesis [14]. *RUNX1* controls the expression of various target genes involved in haematopoietic differentiation [34, 35]. The roles of *RUNX1* in normal haematopoiesis are juxtaposed with high frequencies of *RUNX1* mutations and translocations in leukaemia [36, 37]. *RUNX1* is involved in recurrent chromosomal translocations, such as t (8;21) (*RUNX1*‐*RUNX1T1*) and t (3;21) (*EVI1*‐*RUNX1*) in AML [21]. Besides balanced rearrangements, recurrent intragenic mutations have also been identified in AML, MDS and chronic myelomonocytic leukaemia [36, 38, 39]. *RUNX1* somatic mutations are detected in roughly 15% of adult patients with de novo AML [36]. They are closely associated with older age, male gender and inferior prognosis compared to AML patients without *RUNX1* mutations. In MDS, somatic mutations in *RUNX1* occurs in approximately 10% of patients. These patients had a higher propensity and shorter latency for progression to AML than patients with wild *RUNX1* [40].

Although *RUNX1* is generally considered to be a tumour suppressor, accumulated evidence reveals it plays a central role in leukemogenesis and can act as an oncogene as well [14, 41]. Wild‐type *RUNX1* is required for the development of CBF‐AML, including t (8;21)/*RUNX1*‐*RUNX1T1* and inv [16]/*CBFB‐MYH11* leukaemia, which suggests a delicate balance between wild RUNX1 and RUNX1‐fusion protein contributes to leukaemia cell survival [14]. RUNX1 is also indispensable for MLL‐fusion leukaemia [42, 43]. Moreover, AML harbouring *FLT3*‐ITD has higher levels of RUNX1 [44, 45]. In such a context, upregulated RUNX1 cooperates with FLT3‐ITD to induce leukaemia. From a clinical aspect of view, Morita et al. analysed the OS of AML patients from TCGA clinical datasets (*n* = 187) [46]. Patients were divided into tripartitions according to their *RUNX1* expressions. *RUNX1* intermediately expressing AML patients displayed the worst OS, whereas those with low *RUNX1* expressions exhibited the most favourable prognosis. Concomitantly, another recent study revealed that among patients with CN‐AML, higher *RUNX1* expressions were associated with significantly worse OS in two independent cohorts [20]. Recently, Wesely and his colleagues demonstrated the essential role of RUNX1 in LSCs [19]. They found that reducing the RUNX1 protein by approximately 50% in the LSCs markedly abrogated their ability to engraft while RUNX1 knockdown abolished bulk cell survival and colony formation in primary human AML samples across diverse genetic groups. The authors therefore proposed that RUNX1 may be a therapeutic target in LSC elimination. In accordance with the findings, knocking down RUNX1 inhibited leukaemia cells growth in both human CD34+ cells transduced with MLL‐AF9 and the MLL‐AF9 mouse model [42]. Mill et al. conducted similar studies in *RUNX1* mutant AML and found that Knockdown of RUNX1 inhibited in vitro and in vivo growth of AML and prolonged survival of mice engrafted with mutant RUNX1‐expressing AML [47]. Literature discussing the role of *RUNX1* expression in MDS has been scarce. Herein, we demonstrated that higher *RUNX1* expression significantly predicted poor prognosis in MDS patients through multiple layers of analysis, thereby paving the way for targeting RUNX1 in the treatment of MDS patients with higher *RUNX1* expression.

In this study, higher *RUNX1* expression was shown closely associated with high‐risk characteristics, including excess blasts, poor‐risk karyotypes, higher‐risk IPSS‐R and gene mutations in *ASXL1*, *RUNX1* and *TP53*. Meanwhile, the multivariable analysis revealed higher *RUNX1* expression was an independent poor‐risk factor for LFS and OS. Further, higher *RUNX1* expression could predict shorter LFS and OS in both IPSS‐R higher‐ and lower‐risk patients, as well as *RUNX1*‐mutated and unmutated patients. Collectively, *RUNX1* expression may help identify patients who need more active treatment but are categorized in the low‐risk group by the current risk‐stratification system. For instance, allo‐HSCT could overcome the adverse effect of high *RUNX1* expression in our MDS cohort, suggesting that patients with higher *RUNX1* expression receive allo‐HSCT if eligible.

Notably, in our analysis, higher‐RUNX1 patients could attain a better clinical outcome if they received HSCT. However, the immortal time bias in the retrospective cohort might lead to a biased association. Furthermore, the decision to proceed and the timing of HSCT also varied due to a multitude of reasons, including but not limited to disease stages, availabilities of suitable donors and comorbidities, hence introducing deeper heterogeneity. While a sounder analysis with time‐dependent covariates may help mitigate bias, prospective investigations are required to make a more precise estimate of the impact of HSCT in patients with high *RUNX1* expression.

Bioinformatics analysis showed a close association of higher *RUNX1* expression with CE HSC/LSC signature and various annotated HSC‐ or LSC‐relevant signatures. In the study to establish a LSC score in MDS patients, we previously identified RUNX1, ASXL1 and TP53 mutations correlated with robust LSC character in MDS while SF3B1 mutation, the opposite [11]. The present study showed that *RUNX1*, *ASXL1* or *TP53* mutations were closely associated with higher *RUNX1* expression while *SF3B1* mutations, on the contrary, with lower *RUNX1* expression. From these findings, it is suggested that *RUNX1* is indispensable for LSC maintenance. Meanwhile, the casual relationship between *RUNX1* expression and disease progression, as well as the dynamics and physiologic implications of *RUNX1* expression in the BM, awaits further investigation.

In summary, the investigations herein provide evidence that RUNX1 expression can be prognostic for LFS and OS in patients with MDS, corresponding to that in patients with AML. The prognostic relevance remained valid across IPSS‐R subgroups, among patients with different *RUNX1* mutation statuses, and in two external independent cohorts. Higher expression of *RUNX1* was also confirmed prognostically detrimental in the multivariable analysis. In connection to the above, experimental studies will be needed to foster our understanding of the regulation of *RUNX1* in the heterogeneous cellular contexts of MDS and ultimately deliver patient‐tailored therapeutic avenues.

## CONFLICT OF INTEREST

The authors declare that they have no competing interests.

## ETHIC STATEMENT

The Research Ethics Committee of the NTUH approved this study (approval number: 201709072RINC), and written informed consent was provided according to the Declaration of Helsinki.

## AUTHOR CONTRIBUTIONS

YHW was responsible for data collection and management, statistical analysis, interpretation, visualization, literature research and manuscript writing. CLC, CYY and CLH assisted in statistical analysis. CHT, HAH and WCC were responsible for data collection and management. CCL and HFT conceived and coordinated the study and revised the manuscript.

## Supporting information

Supporting Information

## Data Availability

The data reported in this article can be accessed through reasonable requests from the corresponding authors.
