# Supplementary material for: Higher RUNX1 expression levels are associated with worse overall and leukaemia‐free survival in myelodysplastic syndrome patients
Source: EJHaem. 2022 Aug 19;3(4):1209–19. doi: 10.1002/jha2.547 (PMC9713038; doi:10.1002/jha2.547)
Supplement: Supplementary file 1 — Supporting Information [file JHA2-3-1209-s001.docx]

**Supplemental data**

Supplementary method 1 ~ 2

Supplemental tables: 1 ~ 8

Supplemental figures: 1 ~ 7

**Supplementary method 1 -** **Library preparation and RNA sequencing**

The purified RNA was used to prepare the sequencing library using the TruSeq Stranded mRNA Library Prep Kit (Illumina, San Diego, CA, USA) following the manufacturer’s recommendations. Briefly, mRNA was purified from total RNA (1 μg) using oligo(dT)-coupled magnetic beads and fragmented into small pieces at elevated temperatures. First-strand cDNA was synthesized using reverse transcriptase and random primers. After the generation of double-strand cDNA and adenylation on the 3’ ends of DNA fragments, the adaptors were ligated and purified with the AMPure XP system (Beckman Coulter, Beverly, USA). The quality of the libraries was assessed using an Agilent Bioanalyzer 2100 system and a real-time polymerase chain reaction (PCR) system. The qualified libraries were then sequenced on an Illumina NovaSeq 6000 platform with 150 bp paired-end reads generated by Genomics, BioSci & Tech Co., New Taipei City, Taiwan. The bases with low quality and sequences from adapters in the raw data were removed using Cutadapt (v 3.0). The qualified reads were aligned to the reference genome GRCh38 using STAR (v2.7.6a), and read counts of each gene based on GENCODE (v28) were calculated using featureCounts (v2.0.1). Gene expression levels were normalized to metric transcripts per million (TPM).

**Supplementary method 2 - Statistical analysis**

The Mann-Whitney U and Kruskal–Wallis tests were applied to compare medians and continuous variables of distribution. Fisher’s exact test or χ^2^ test was used to examine differences among discrete variables. Pearson’s correlation coefficient (PCC) was calculated to estimate correlations of variables where relevant. The median value of *RUNX1* expression was calculated to compare clinical features and outcomes of patients with expression values above or below the median. Survival analyses were evaluated by the Kaplan-Meier method. Relative risks were presented as hazard ratios (HR) adjusted for disease modifying therapies (hypomethylation agents and allogeneic hematopoeitic stem cell transplantation) and confounding factors where relevant. LFS was defined as the duration from the date of diagnosis to the last follow-up, documented acute leukaemia transformation, or death from any cause, whichever occurred first. OS was defined as the duration from diagnosis to the last follow-up or death from any cause, whichever came first. The Cox proportional hazard model was used for univariate and backward conditional multivariable analyses. Sensitivity analysis with random effects was used to assess the homogeneity of results across the three cohorts. All tests were 2-sided and were considered statistically significant if *p*<0.05. All analyses were conducted using Review Manager 5.3, IBM SPSS Statistics 23 for Windows and R software (version 4.0.3).

**Supplemental Table 1. Full list of 54 myeloid neoplasm-relevant genes in the study of targeted NGS sequencing.**

| **Gene name** | **Target region (exon)** | **Gene name** | **Target region (exon)** |
| --- | --- | --- | --- |
| ***ABL*** | 4-6 | ***JAK3*** | 13 |
| ***ASXL1*** | 12 | ***KDM6A*** | full |
| ***ATRX*** | 8-10 and 17-31 | ***KIT*** | 2, 8-11, 13+17 |
| ***BCOR*** | full | ***KRAS*** | 2+3 |
| ***BCORL1*** | full | ***MLL*** | 5-8 |
| ***BRAF*** | 15 | ***MPL*** | 10 |
| ***CALR*** | 9 | ***MYD88*** | 3-5 |
| ***CBL*** | 8+9 | ***NOTCH1*** | 26-28, 34 |
| ***CBLB*** | 9, 10 | ***NPM1*** | 12 |
| ***CBLC*** | 9, 10 | ***NRAS*** | 2+3 |
| ***CDKN2A*** | full | ***PDGFRA*** | 12, 14, 18 |
| ***CEBPA*** | full | ***PHF6*** | full |
| ***CSF3R*** | 14-17 | ***PTEN*** | 5+7 |
| ***CUX1*** | full | ***PTPN11*** | 3+13 |
| ***DNMT3A*** | full | ***RAD21*** | full |
| ***ETV6*** | full | ***RUNX1*** | full |
| ***EZH2*** | full | ***SETBP1*** | 4 (partial) |
| ***FBXW7*** | 9+10+11 | ***SF3B1*** | 13-16 |
| ***FLT3*** | 14+15+20 | ***SMC1A*** | 2, 11, 16+17 |
| ***GATA1*** | 2 | ***SMC3*** | 10, 13, 19, 23, 25+28 |
| ***GATA2*** | 2-6 | ***SRSF2*** | 1 |
| ***GNAS*** | 8+9 | ***STAG2*** | full |
| ***HRAS*** | 2+3 | ***TET2*** | 3-11 |
| ***IDH1*** | 4 | ***TP53*** | 2-11 |
| ***IDH2*** | 4 | ***U2AF1*** | 2+6 |
| ***IKZF1*** | full | ***WT1*** | 7+9 |
| ***JAK2*** | 12+14 | ***ZRSR2*** | full |

**Supplemental Table 2. Mutation pattern and expression of *RUNX1* in patients with mutant *RUNX1***

| **Patient** | **Mutation** | **Terminal** | **Allele**  **frequency (%)** | **Expression (TPM)** | **Patient** | **Mutation** | **Terminal** | **Allele frequency (%)** | **Expression (TPM)** |
| --- | --- | --- | --- | --- | --- | --- | --- | --- | --- |
| 17 | p.Asn146ThrfsTer17 | N | 21.5 | 45.34 | 81 | p.Leu210Ter | C | 12.9 | 93.55 |
| 24 | p.Arg107His | N | 40.3 | 70.16 | 84 | p.Tyr414Ter | C | 30.3 | 108.87 |
| 50 | p.Asn96GlnfsTer42 | N | 30.3 | 55.26 | 91 | p.Pro384LeufsTer220 | C | 19.3 | 97.25 |
| 61 | p.Asp62GlyfsTer76 | N | 40.2 | 86.11 | 96 | p.Arg204Gln | C | 34 | 145.98 |
| 118 | p.Phe97GlnfsTer42 | N | 36.5 | 116.92 | 98 | p.Arg346ProfsTer254 | C | 43.7 | 228.01 |
| 121 | p.Ser167_Gly168del | N | 40.7 | 81.75 | 112 | p.Arg320Ter | C | 18.06 | 119.89 |
| 135 | p.Ser141Leu | N | 49.8 | 123.49 | 114 | p.Tyr377Ter | C | 18.5 | 77.25 |
| 140 | p.Leu161Pro | N | 36.4 | 71.88 | 117 | p.Tyr287SerfsTer314 | C | 21.61 | 70.65 |
| 148 | p.Leu175Pro | N | 40.4 | 57.06 | 123 | p.Arg204Gln | C | 48 | 127.92 |
| 165 | p.Gly69AlafsTer3 | N | 21.5 | 146.88 | 127 | p.Glu422ArgfsTer179 | C | 25.1 | 189.86 |
| 197 | p.Arg107Cys | N | 14.9 | 85.51 | 131 | p.Arg320Ter | C | 21.6 | 83.26 |
| 231 | p.Arg107Pro | N | 22.3 | 103.62 | 142 | p.Met368HisfsTer232 | C | 15.28 | 106.56 |
| 237 | p.Asn153GlufsTer7 | N | 57.5 | 81.19 | 143 | p.Gln335His | C | 49 | 57.54 |
| 252 | p.Val155ProfsTer6 | N | 6.1 | 55.46 | 160 | p.Tyr349Ter | C | 9.5 | 59.24 |
| 292 | p.Asn139LysfsTer5 | N | 40 | 65.99 | 162 | p.Arg201Gln | C | 39.8 | 115.13 |
| 1 | p.Ile364ValfsTer231 | C | 39.9 | 26.75 | 171 | p.Ser318PhefsTer282 | C | 25.6 | 95.43 |
| 7 | p.Gln397Arg | C | 45.6 | 15.47 | 187 | p.Asp332Asn | C | 50.4 | 60.14 |
| 14 | p.Gln397ProfsTer203 | C | 39 | 100.71 | 207 | p.Pro261Arg | C | 51.7 | 31.35 |
| 22 | c.805+2T>A | C | 50.3 | 87.04 | 246 | p.Arg376ProfsTer224 | C | 5 | 61.00 |
| 32 | p.Arg346ProfsTer254 | C | 32.2 | 85.59 | 250 | p.Gln272Ter | C | 39.26 | 54.53 |
| 48 | p.Phe396ValfsTer204 | C | 25.8 | 90.30 | 304 | p.Arg201Gln | C | 30.3 | 31.83 |
| 51 | p.Arg191SerfsTer21 | C | 24.5 | 32.10 | 309 | p.Gln390Ter | C | 42.8 | 85.27 |
| 53 | p.Pro350SerfsTer250 | C | 43.3 | 110.47 | 311 | p.Ser388PhefsTer212 | C | 48.16 | 111.94 |
| 62 | p.Tyr380ProfsTer219 | C | 25.7 | 111.39 | 318 | p.Gly199Arg | C | 40.98 | 118.86 |
| 71 | p.Val179Asp | C | 37.1 | 155.56 | 324 | p.Gln213ArgfsTer24 | C | 29.64 | 131.88 |
| 75 | p.Pro357GlyfsTer245 | C | 21.1 | 158.52 | 337 | p.Gln213SerfsTer15 | C | 48.31 | 84.00 |
| 79 | p.Ala409LeufsTer190 | C | 7.3 | 104.39 | 340 | p.Glu474GlyfsTer121 | C | 25.82 | 104.30 |

**Supplemental Table 3. Comparison of cytogenetic changes between patients with lower and higher *RUNX1* expression**

| Variables | Total*  (n=332) | Lower *RUNX1* (n=165)* | Higher *RUNX1*  (n=167)* | *P* value |
| --- | --- | --- | --- | --- |
| Normal karyotype | 185 (55.7) | 97 (58.8) | 88 (52.7) | 0.271 |
| Any abnormality |  |  |  |  |
| Loss Y^†^ | 11 (3.3) | 7 (4.2) | 4 (2.4) | 0.377 |
| Del 20q^†^ | 14 (4.2) | 9 (5.5) | 5 (3.0) | 0.289 |
| Del 5q^†^ | 2 (0.6) | 1 (0.6) | 1 (0.6) | >0.999 |
| Trisomy 8^†^ | 26 (7.8) | 13 (7.9) | 13 (7.9) | >0.999 |
| Poor and very poor risk groups | 51 (15.4) | 12 (7.3) | 39 (23.4) | <0.001 |
| -7, inv(3)/t(3q)/del(3q),  double including -7/del(7q) | 7 (2.1) | 1 (0.6) | 6 (3.6) | 0.121 |
| Complex karyotype^‡^ | 44 (13.3) | 11 (6.7) | 33 (19.8) | 0.001 |
| Other abnormalities | 43 (13.0) | 26 (15.8) | 17 (10.3) | 0.144 |

*Cytogenetic data at diagnosis were available in 332 patients, including 165 with lower RUNX1 expression and 167 with higher RUNX1 expression

^†^As the sole abnormality.

**^‡^**Three or more abnormalities

**Supplemental Table 4. Comparison of gene mutations between patients with lower and higher *RUNX1* expression**

| Genes | Total (n=333) | Lower *RUNX1* (n=167) | Higher *RUNX1* (n=166) | *P* value |
| --- | --- | --- | --- | --- |
| *ABL* | 1 (0.3) | 0 (0) | 1 (0.6) | 0.498 |
| *ASXL1* | 83 (24.9) | 29 (17.4) | 54 (32.5) | 0.002 |
| *BCOR* | 20 (6.0) | 7 (4.2) | 13 (7.8) | 0.175 |
| *BCORL1* | 3 (0.9) | 1 (0.6) | 2 (1.2) | 0.623 |
| *CALR* | 1 (0.3) | 1 (0.6) | 0 (0) | >0.999 |
| *CBL* | 10 (3.0) | 4 (2.4) | 6 (3.6) | 0.542 |
| *CEBPA* | 13 (3.9) | 7 (4.2) | 6 (3.6) | >0.999 |
| *CUX1* | 5 (1.5) | 3 (1.8) | 2 (1.2) | >0.999 |
| *DNMT3A* | 40 (12.0) | 17 (10.2) | 23 (13.9) | 0.317 |
| *ETV6* | 9 (2.7) | 4 (2.4) | 5 (3.0) | 0.750 |
| *EZH2* | 20 (6.0) | 6 (3.6) | 14 (8.4) | 0.069 |
| *FLT3*-ITD | 2 (0.6) | 0 (0) | 2 (1.2) | 0.248 |
| *GATA2* | 8 (2.4) | 3 (1.8) | 5 (3.0) | 0.502 |
| *GNAS* | 2 (0.6) | 1 (0.6) | 1 (0.6) | >0.999 |
| *IDH1* | 2 (0.6) | 0 (0) | 2 (1.2) | 0.248 |
| *IDH2* | 14 (4.2) | 6 (3.6) | 8 (4.8) | 0.599 |
| *IKZF1* | 2 (0.6) | 1 (0.6) | 1 (0.6) | >0.999 |
| *JAK2* | 2 (0.6) | 0 (0) | 2 (1.2) | 0.248 |
| *KDM6A* | 2 (0.6) | 0 (0) | 2 (1.2) | 0.248 |
| *KIT* | 3 (0.9) | 1 (0.6) | 2 (1.2) | 0.623 |
| *KRAS* | 5 (1.5) | 1 (0.6) | 4 (2.4) | 0.215 |
| *MLL* | 6 (1.8) | 2 (1.2) | 4 (2.4) | 0.448 |
| *MPL* | 2 (0.6) | 0 (0) | 2 (1.2) | 0.248 |
| *NOTCH1* | 1 (0.3) | 1 (0.6) | 0 (0) | >0.999 |
| *NPM1* | 12 (3.6) | 1 (0.6) | 11 (6.6) | 0.003 |
| *NRAS* | 21 (6.3) | 7 (4.2) | 14 (8.4) | 0.121 |
| *PHF6* | 7 (2.1) | 3 (1.8) | 4 (2.4) | 0.723 |
| *PTPN11* | 6 (1.8) | 2 (1.2) | 4 (2.4) | 0.448 |
| *RAD21* | 2 (0.6) | 0 (0) | 2 (1.2) | 0.248 |
| *RUNX1* | 54 (16.2) | 14 (8.4) | 40 (24.1) | <0.001 |
| *SETBP1* | 9 (2.7) | 4 (2.4) | 5 (3.0) | 0.750 |
| *SF3B1* | 49 (14.7) | 35 (21.0) | 14 (8.4) | 0.002 |
| *SMC1A* | 1 (0.3) | 0 (0) | 1 (0.6) | 0.498 |
| *SMC3* | 1 (0.3) | 0 (0) | 1 (0.6) | 0.498 |
| *SRSF2* | 33 (9.9) | 7 (4.2) | 26 (15.7) | <0.001 |
| *STAG1* | 1 (0.3) | 1 (0.6) | 0 (0) | >0.999 |
| *STAG2* | 42 (12.6) | 15 (9.0) | 27 (16.3) | 0.049 |
| *TET2* | 46 (13.8) | 15 (9.0) | 31 (18.7) | 0.028 |
| *TP53* | 43 (12.9) | 11 (6.6) | 32 (19.3) | 0.001 |
| *U2AF1* | 26 (7.8) | 15 (9.0) | 11 (6.6) | 0.541 |
| *WT1* | 4 (1.2) | 1 (0.6) | 3 (1.8) | 0.371 |
| *ZRSR2* | 17 (5.1) | 4 (2.4) | 13 (7.8) | 0.027 |

Data are presented as n (%).

*P* values of <0.05 are statistically significant.

**Supplemental Table 5. Tests of different variables as potential confounding factors with *RUNX1* expression for overall survival**

|  | **Variable** | **HR** | **Lower 95% CI** | **Upper 95% CI** | ***P* value** | **Change of HR (%)** |
| --- | --- | --- | --- | --- | --- | --- |
| **Higher *RUNX1* expression** | alone | 2.360 | 1.706 | 3.264 | <0.001 | - |
| with | Age | 2.349 | 1.697 | 3.252 | <0.001 | 0.5 |
|  | Sex | 2.363 | 1.708 | 3.268 | <0.001 | -0.1 |
|  | IPSS-R | 1.736 | 1.232 | 2.447 | 0.002 | 26.4 |
|  | Platelet | 2.297 | 1.658 | 3.183 | <0.001 | 2.7 |
|  | Excess of blasts | 1.768 | 1.260 | 2.482 | 0.001 | 25.1 |
|  | Poor-risk karyotypes* | 2.053 | 1.462 | 2.881 | <0.001 | 13.0 |
|  | Supportive care | 2.216 | 1.572 | 3.125 | <0.001 | 6.1 |
|  | Hypomethylation agents | 2.313 | 1.657 | 3.230 | <0.001 | 2.0 |
|  | LDAraC | 2.229 | 1.606 | 3.092 | <0.001 | 5.6 |
|  | Intensive chemotherapies | 2.338 | 1.674 | 3.265 | <0.001 | 1.0 |
|  | HSCT | 2.491 | 1.796 | 3.455 | <0.001 | -5.5 |
|  | *ASXL1* | 2.429 | 1.731 | 3.406 | <0.001 | -2.9 |
|  | *EZH2* | 2.443 | 1.749 | 3.413 | <0.001 | -3.5 |
|  | *NPM1* | 2.465 | 1.760 | 3.452 | <0.001 | -4.4 |
|  | *RUNX1* mutation | 2.396 | 1.707 | 3.363 | <0.001 | -1.5 |
|  | *SF3B1* | 2.356 | 1.685 | 3.292 | <0.001 | 0.2 |
|  | *SRSF2* | 2.418 | 1.718 | 3.403 | <0.001 | -2.5 |
|  | *STAG2* | 2.388 | 1.706 | 3.343 | <0.001 | -1.2 |
|  | *TET2* | 2.436 | 1.742 | 3.406 | <0.001 | -3.2 |
|  | *TP53* | 2.078 | 1.474 | 2.928 | <0.001 | 11.9 |
|  | *ZRSR2* | 2.360 | 1.706 | 3.264 | <0.001 | -5.0 |

*Poor-risk karyotypes: -7, inv(3)/t(3q)/del(3q), double including -7/del(7q), and complex

^†^low-dose cytarabine: at 20 mg once or twice daily for 10 consecutive days every 4–6 weeks.

Abbreviations: HR, hazard ratio; LDAraC, low-dose cytarabine; HSCT, allogeneic hematopoietic stem cell transplantation

**Supplemental table 6. Multivariable analysis for LFS and OS in the 324 MDS patients who had both cytogenetic data and NGS mutation data at diagnosis**

|  | LFS | | | | OS | | | | |
| --- | --- | --- | --- | --- | --- | --- | --- | --- | --- |
|  | **95% CI** | | | | **95% CI** | | | | |
| Variable | **aHR** | **Lower** | **Upper** | ***P*** | **aHR** | **Lower** | **Upper** | ***P*** | |
| Age* | 1.021 | 1.009 | 1.032 | 0.001 | 1.032 | 1.02 | 1.045 | <0.001 | |
| Sex (reference: female) | 1.232 | 0.889 | 1.707 | 0.210 | 1.435 | 1.01 | 2.04 | 0.044 | |
| IPSS-R^†^ | 1.29 | 1.176 | 1.416 | <0.001 | 1.367 | 1.239 | 1.507 | <0.001 | |
| *TP53* | 2.383 | 1.568 | 3.621 | <0.001 | 4.673 | 3.009 | 7.258 | <0.001 |  |
| Higher *RUNX1* expression^*^ | 1.009 | 1.005 | 1.014 | <0.001 | 1.007 | 1.002 | 1.012 | 0.004 | |

Abbreviations: aHR, adjusted hazard ratios; CI, confidence interval.

Statistically significant if *P*<0.05.

^*^Age, as a continuous variable analysis.

^†^IPSS-R risk groups: Very good, good, intermediate, poor, very poor

**Supplemental table 7. Comparison of clinical and laboratory features between patients with lower and higher *RUNX1* expression in the GSE 114922 cohort**

| **Clinical characters** | **Total (n=78)** | **Lower *RUNX1* (n=39)** | **Higher *RUNX1* (n=39)** | ***P* value** |
| --- | --- | --- | --- | --- |
| **Sex** |  |  |  | >0.999 |
| **Female** | 33 (42.3) | 17 (43.6) | 16 (41.0) |  |
| **Male** | 45 (57.7) | 22 (56.4) | 23 (59.0) |  |
| **Age**^a^ | 67 (26-87) | 65 (26-87) | 70 (34-83) | 0.174 |
| **Laboratory data*** |  |  |  |  |
| **WBC, X 10^9^ /L** | 4.0 (0-30) | 4.74 (1-30) | 3.47 (0-9) | 0.521 |
| **ANC, X 10^9^ /L** | 2.23 (0-20) | 2.64 (0-20) | 1.64 (0-6) | 0.169 |
| **Hb, g/dL** | 9.5 (5-13) | 9 (5-12) | 9.7 (5-13) | 0.172 |
| **Platelet, X 10^9^ /L** | 166 (16-594) | 226 (43-594) | 113 (16-504) | 0.066 |
| **BM blast (%)** | 4 (0-18) | 3 (0-15) | 7 (0-18) | 0.001 |
| **2008 WHO classification** |  |  |  | 0.001 |
| **RA** | 19 (24.4) | 10 (25.6) | 9 (23.1) |  |
| **RARS** | 29 (37.2) | 22 (56.4) | 7 (17.9) |  |
| **RAEB** | 30 (38.5) | 7 (17.9) | 23 (59.0) |  |
| **IPSS-R**^†,‡^ |  |  |  | 0.01 |
| **Very low** | 11 (14.7) | 6 (15.8) | 5 (13.5) |  |
| **Low** | 28 (37.3) | 21 (55.3) | 7 (18.9) |  |
| **Intermediate** | 19 (25.3) | 7 (18.4) | 12 (32.4) |  |
| **High** | 11 (14.7) | 3 (7.9) | 8 (21.6) |  |
| **Very high** | 6 (8.0) | 1 (2.6) | 5 (13.5) |  |
| **IPSS-R lower-risk**^§^ | 58 (77.3) | 34 (89.5) | 24 (64.9) | 0.014 |
| **IPSS-R higher-risk**^§^ | 17 (22.7) | 4 (10.5) | 13 (35.1) |  |

Statistically significant if *P* value < 0.05

*Median (range).

†75 patients, including 38 with lower *RUNX1* expression and 37 with *higher* RUNX1 expression, had comprehensive data for IPSS-R classification at diagnosis.

^‡^IPSS-R: Very low, ≦1.5; Low, >1.5-3; intermediate,>3-4.5; High, >4.5-6; and Very high, >6.

^§^IPSS-R lower-risk: Very low, low, and intermediate; IPSS-R higher-risk: high and very high

Abbreviations: ANC, absolute neutrophil count; Hb, hemoglobin; RA, refractory anemia; RARS, refractory anemia with ring sideroblasts; RAEB, refractory anemia with excess blasts; IPSS-R, revised international prognosis scoring system

**Supplemental table 8. Comparison of clinical and laboratory features between patients with lower and higher *RUNX1* expression in the GSE 15061 cohort**

| **Clinical characters** | **Total**  **(n=110)** | **Lower *RUNX1***  **(n=55)** | **Higher *RUNX1***  **(n=55)** | ***P* value** |
| --- | --- | --- | --- | --- |
| **Age*** | 74 (18-89) | 72 (18-88) | 75 (33-89) | 0.125 |
| **2008 WHO classification** |  |  |  | 0.001 |
| **RA** | 35 (31.8) | 9 (16.4) | 26 (47.3) |  |
| **RARS** | 20 (18.2) | 15 (27.3) | 5 (9.1) |  |
| **5q- syndrome** | 10 (9.1) | 7 (12.7) | 3 (5.5) |  |
| **RCMD** | 7 (6.4) | 6 (10.9) | 1 (1.8) |  |
| **RAEB** | 38 (34.5) | 18 (32.7) | 20 (36.4) |  |
| **IPSS**^†^ |  |  |  | 0.373 |
| **Low** | 53 (48.2) | 27 (49.1) | 26 (47.3) |  |
| **Intermediate-1** | 32 (29.1) | 19 (34.5) | 13 (23.6) |  |
| **Intermediate-2** | 19 (17.3) | 7 (12.7) | 12 (21.8) |  |
| **High** | 6 (5.5) | 2 (3.6) | 4 (7.3) |  |
| **IPSS lower-risk**^‡^ | 85 (77.3) | 46 (83.6) | 39 (70.9) | 0.172 |
| **IPSS higher-risk**^‡^ | 25 (22.7) | 9 (16.4) | 16 (29.1) |  |
| **Karyotypes**^§^ |  |  |  |  |
| **Normal karyotype or 1 of -Y, del(5q), del(20q)** | 91 (82.7) | 44 (80.0) | 47 (85.5) | 0.615 |
| **Intermediate** | 11 (10.0) | 7 (12.7) | 4 (7.3) | 0.527 |
| **Poor-risk karyotype** | 8 (7.3) | 4 (7.3) | 4 (7.3) | >0.999 |

*Median (range).

^†^IPSS: Low, 0; intermediate-1, 0.5-1; intermediate-2, 1.5-2; High, ≥2.5

^‡^IPSS lower-risk: Low and intermediate-1; IPSS higher-risk: intermediate-2 and high

^§^Poor-risk: complex (≧3 abnormalities) or chromosome 7 anomalies; intermediate: other abnormalities

Abbreviations: ANC, absolute neutrophil count; Hb, hemoglobin; IPSS, international prognosis scoring system; RA, refractory anemia; RARS, refractory anemia with ring sideroblasts; RAEB, refractory anemia with excess blasts; RCMD, refractory cytopenia with multilineage dysplasia

**Supplemental Figure 1. Histogram plots and box plots depicting *RUNX1* expression in the entire cohort, different IPSS-R risk groups, and patients with different *RUNX1* mutation statuses and mutation sites.**

(a) The histogram of *RUNX1* expression in the entire cohort.

(b) The *RUNX1* expression were significantly correlated with IPSS-R with differential distribution among IPSS-R subgroups.

(c) The *RUNX1* expression were differentially distributed among patients with different *RUNX1* mutation statuses and mutation sites.

| **a** | **b** | **c** |
| --- | --- | --- |
| 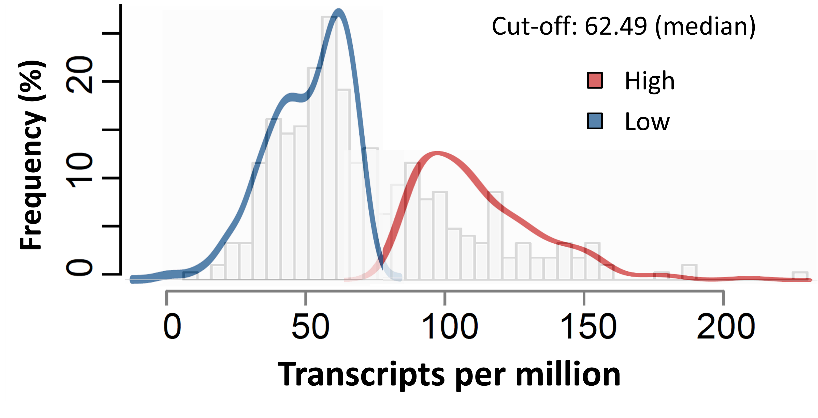 | 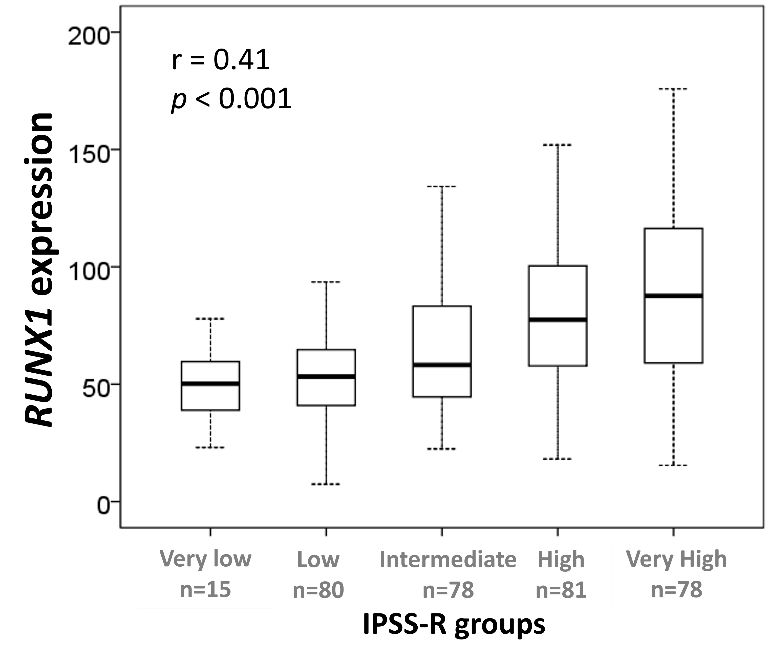 | 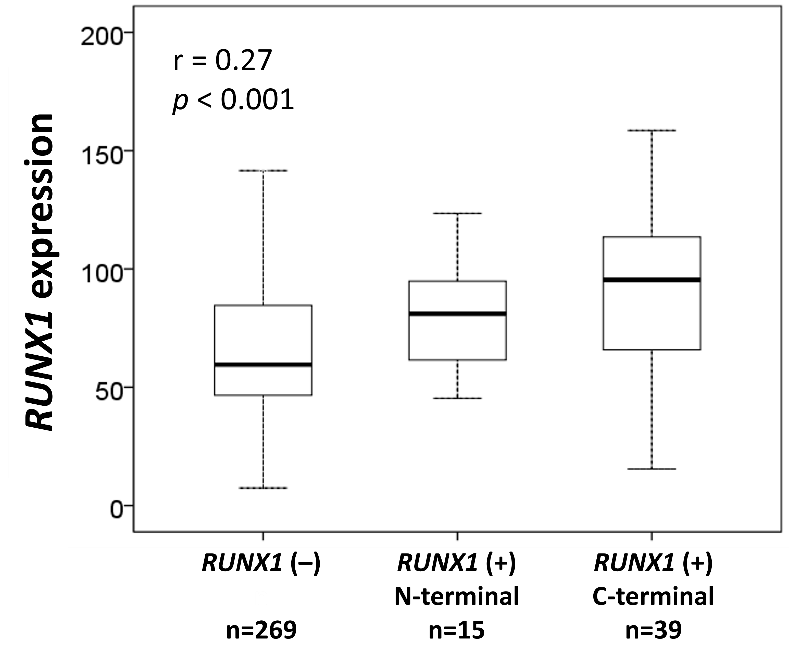 |

**Supplemental Figure 2. Kaplan-Meier survival curves stratified by mutation status of *RUNX1*.**

(a) LFS and (b) OS of the 333 patients stratified by the *RUNX1* mutation status. Patients with mutated *RUNX1* had worse clinical outcomes than those without *RUNX1* mutation.

| **a** | **b** |
| --- | --- |
| **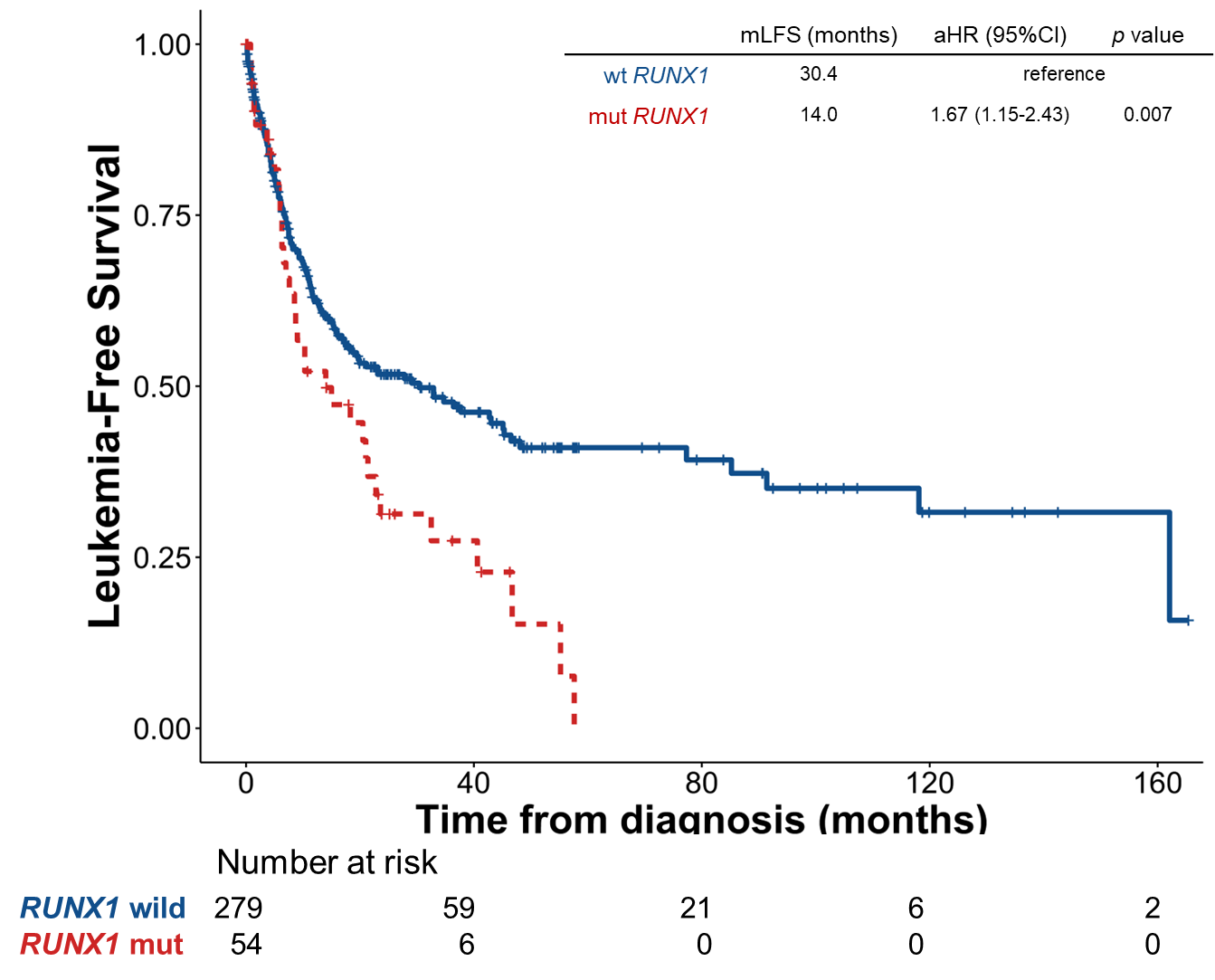** | **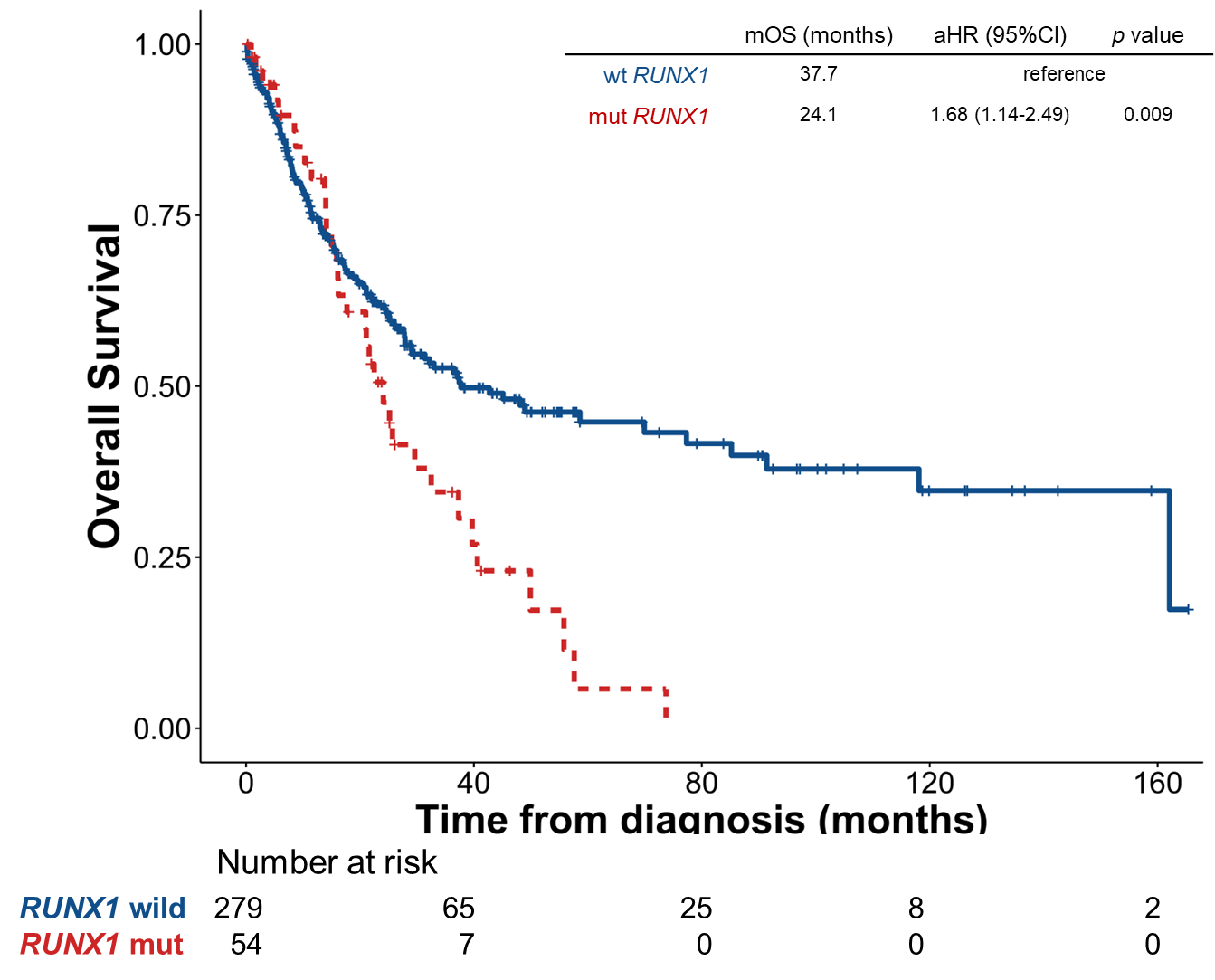** |

**Supplemental Figure 3. Kaplan-Meier plots stratified by *RUNX1* expression in the patients with different karyotypes.** Outcome of the 332 patients who had cytogenetic data at diagnosis. (a) LFS and (b) OS of the 185 patients with normal karyotype; and (c) LFS and (d) OS of the 281 patients without unfavorable cytogenetic changes. Patients with higher *RUNX1* expression had worse clinical outcomes than those with lower expression in patients with different karyotypes.

| **a** | **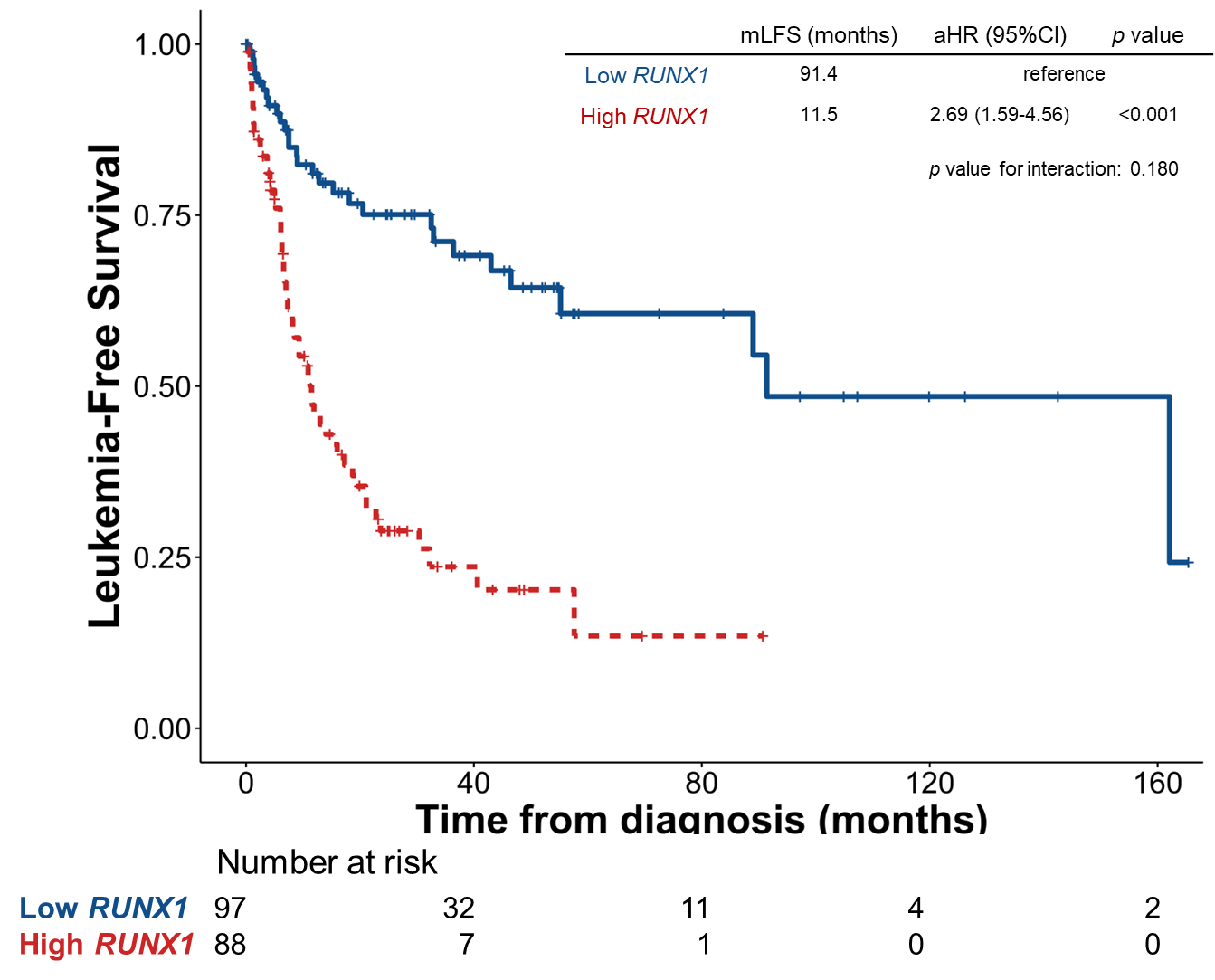** | **b** | **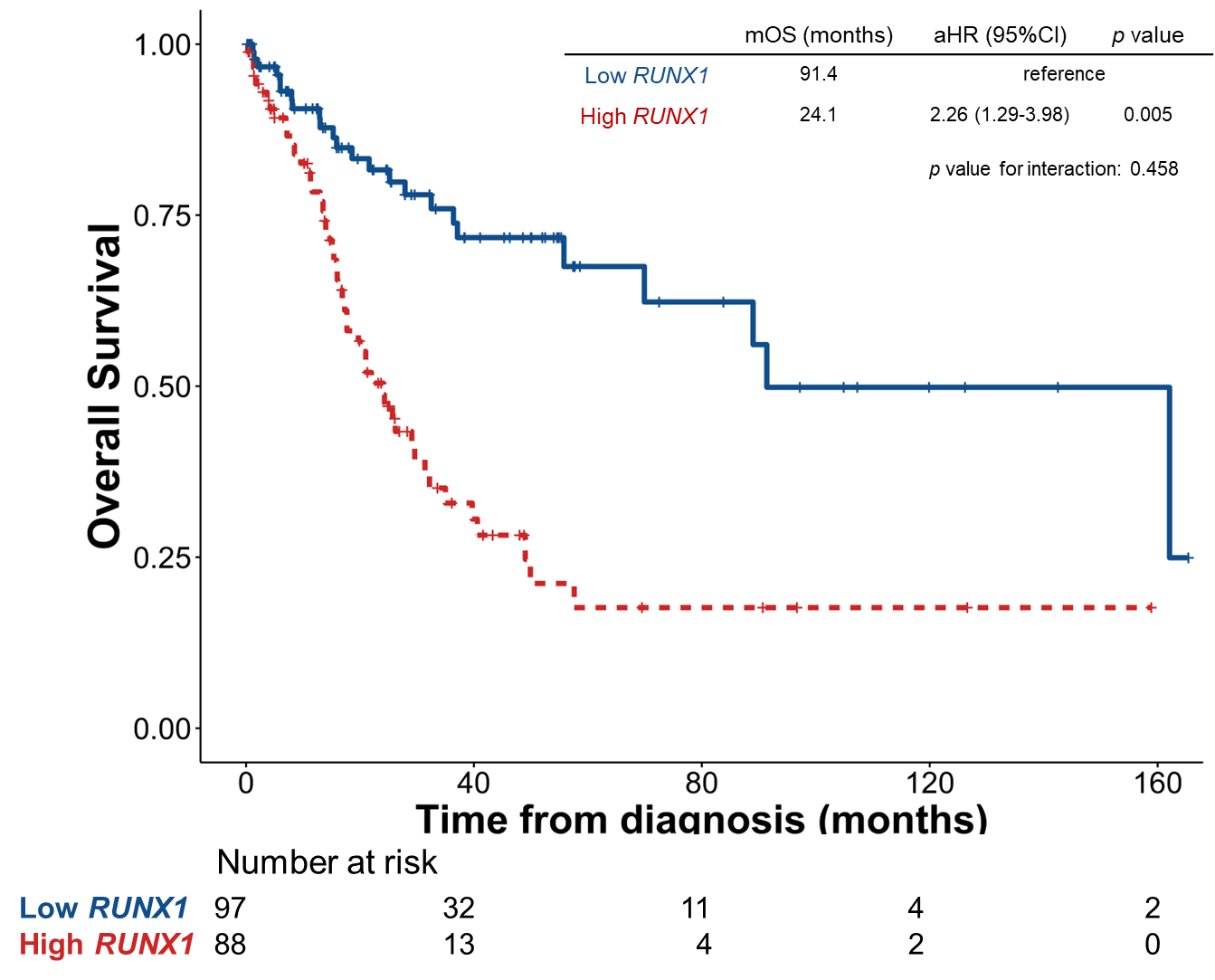** |
| --- | --- | --- | --- |
| **c** | **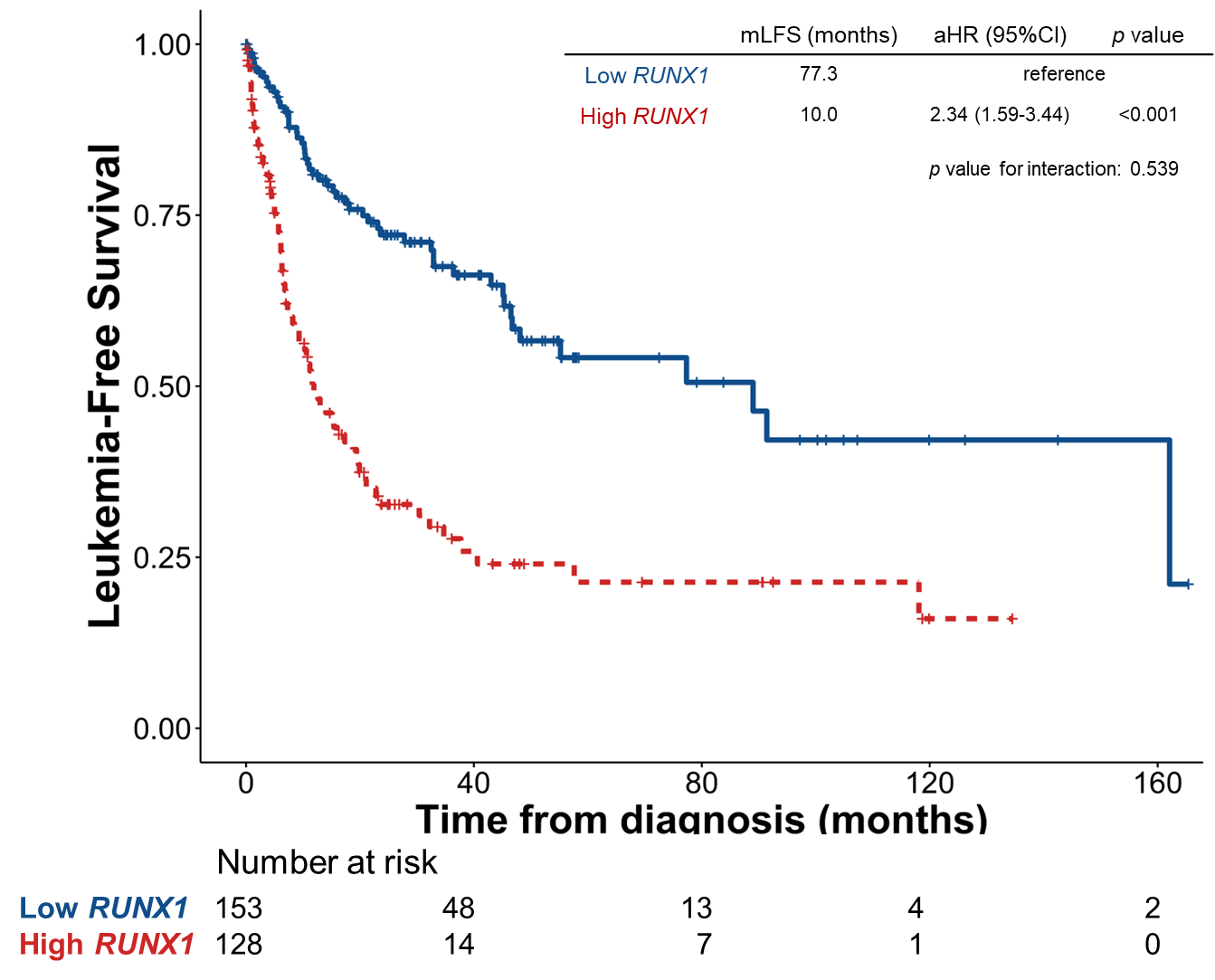** | **d** | **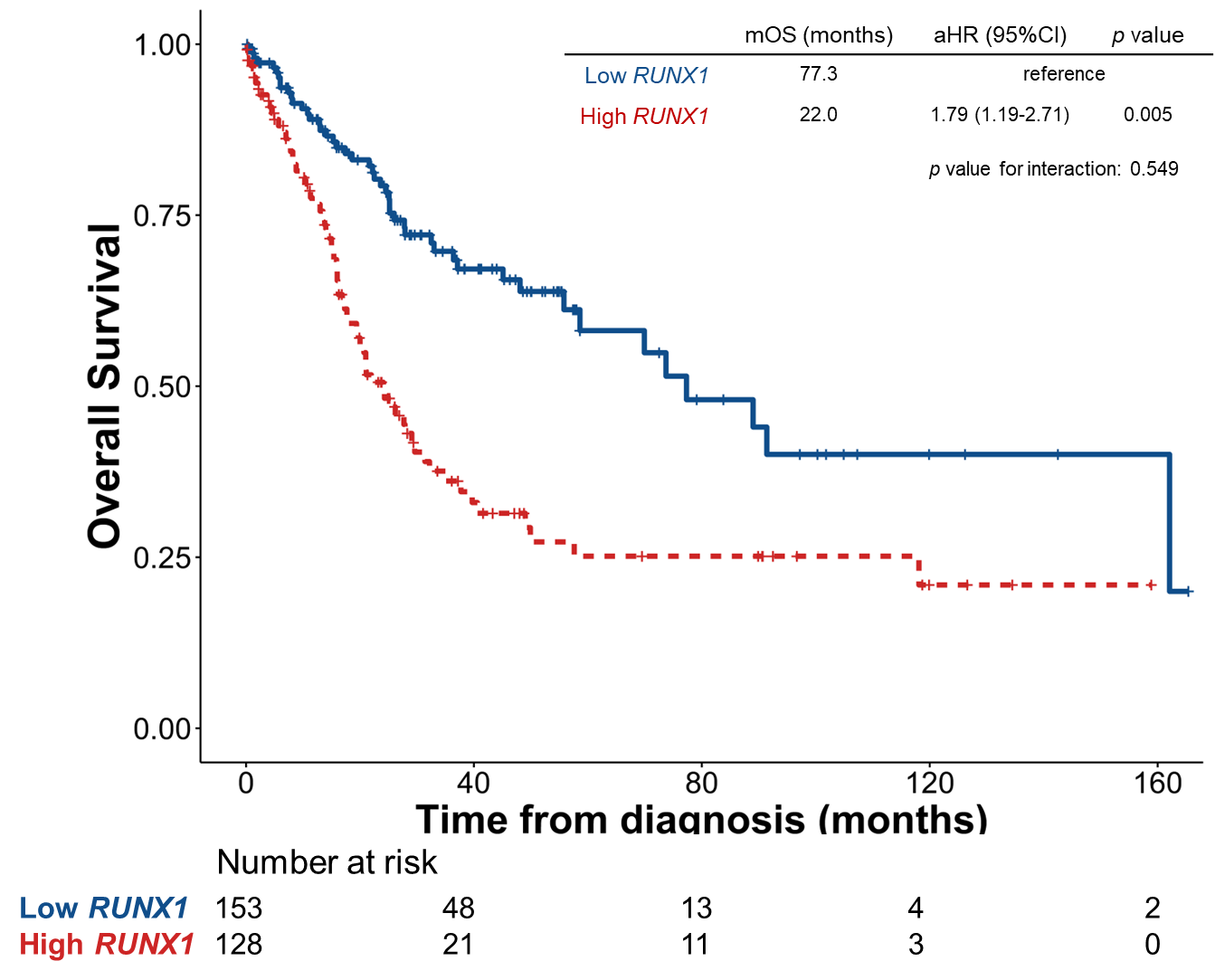** |

**Supplemental Figure 4. Kaplan-Meier plots stratified by *RUNX1* expression in the subgroups of patients receiving different treatment.** (a) LFS and (b) OS of the 164 patients who received supportive care; and (c) LFS and (d) OS of the 177 patients who received active treatment. Higher *RUNX1* expression predicted worse clinical outcomes in both groups.

| **a** | **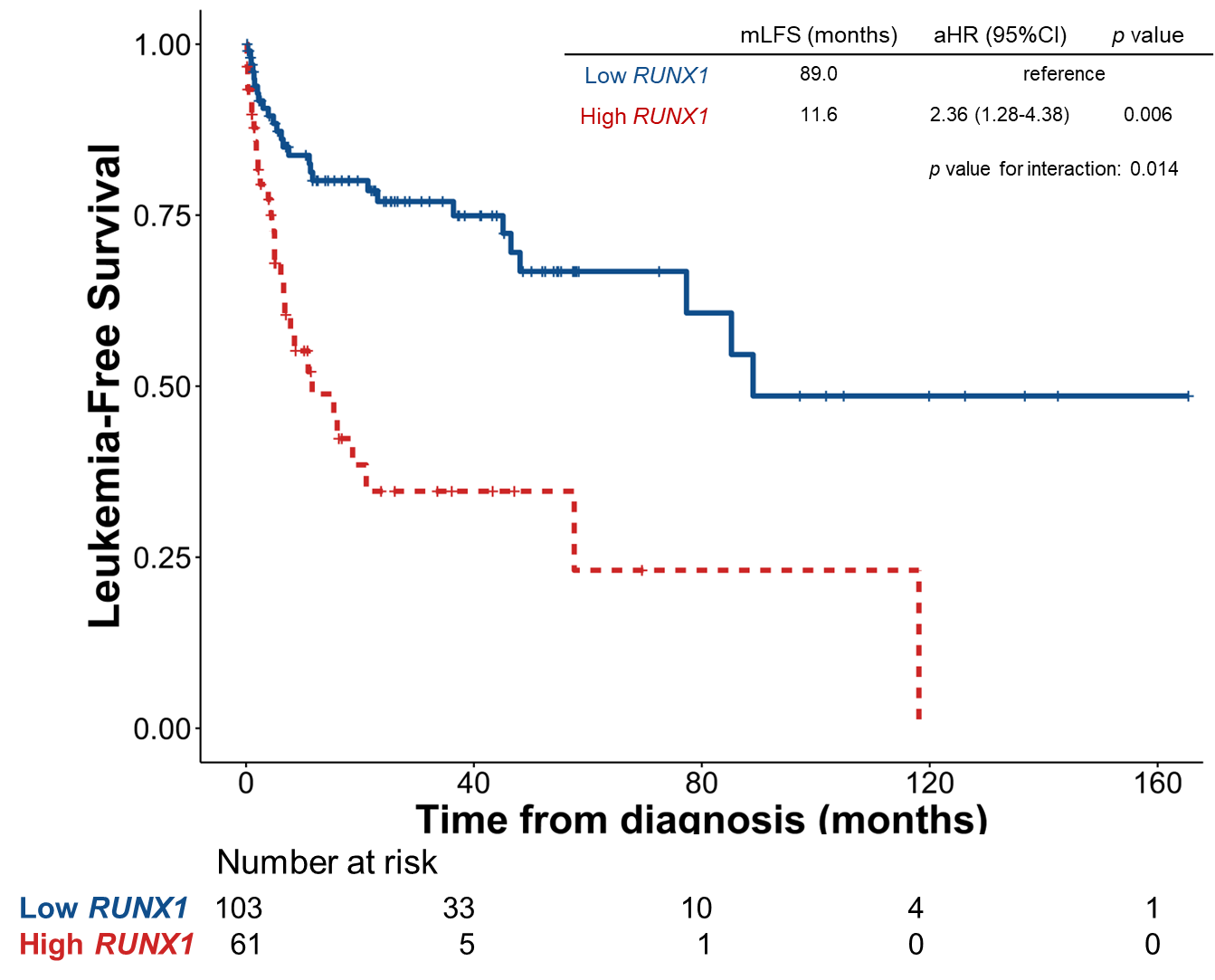** | **b** | **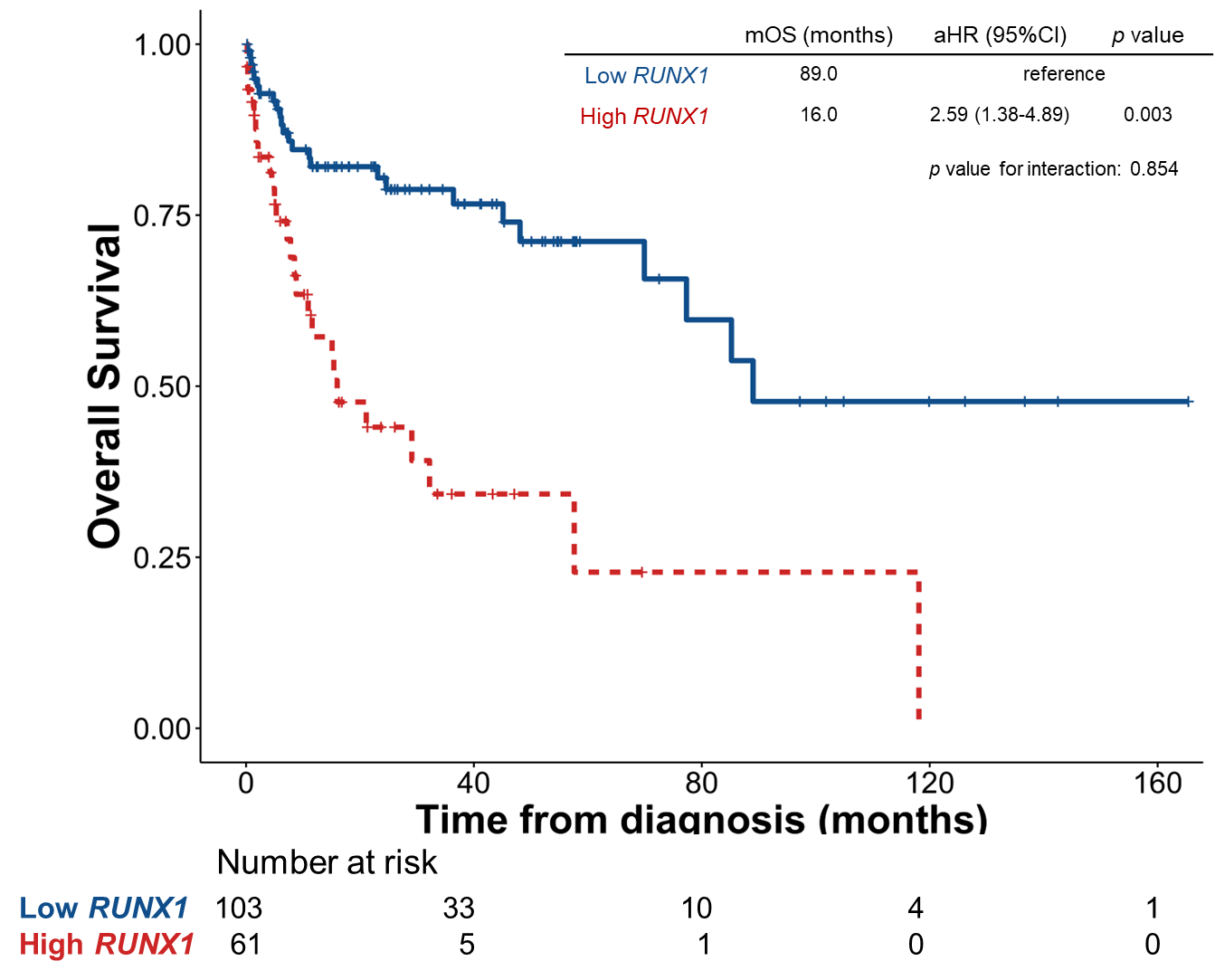** |
| --- | --- | --- | --- |
| **c** | **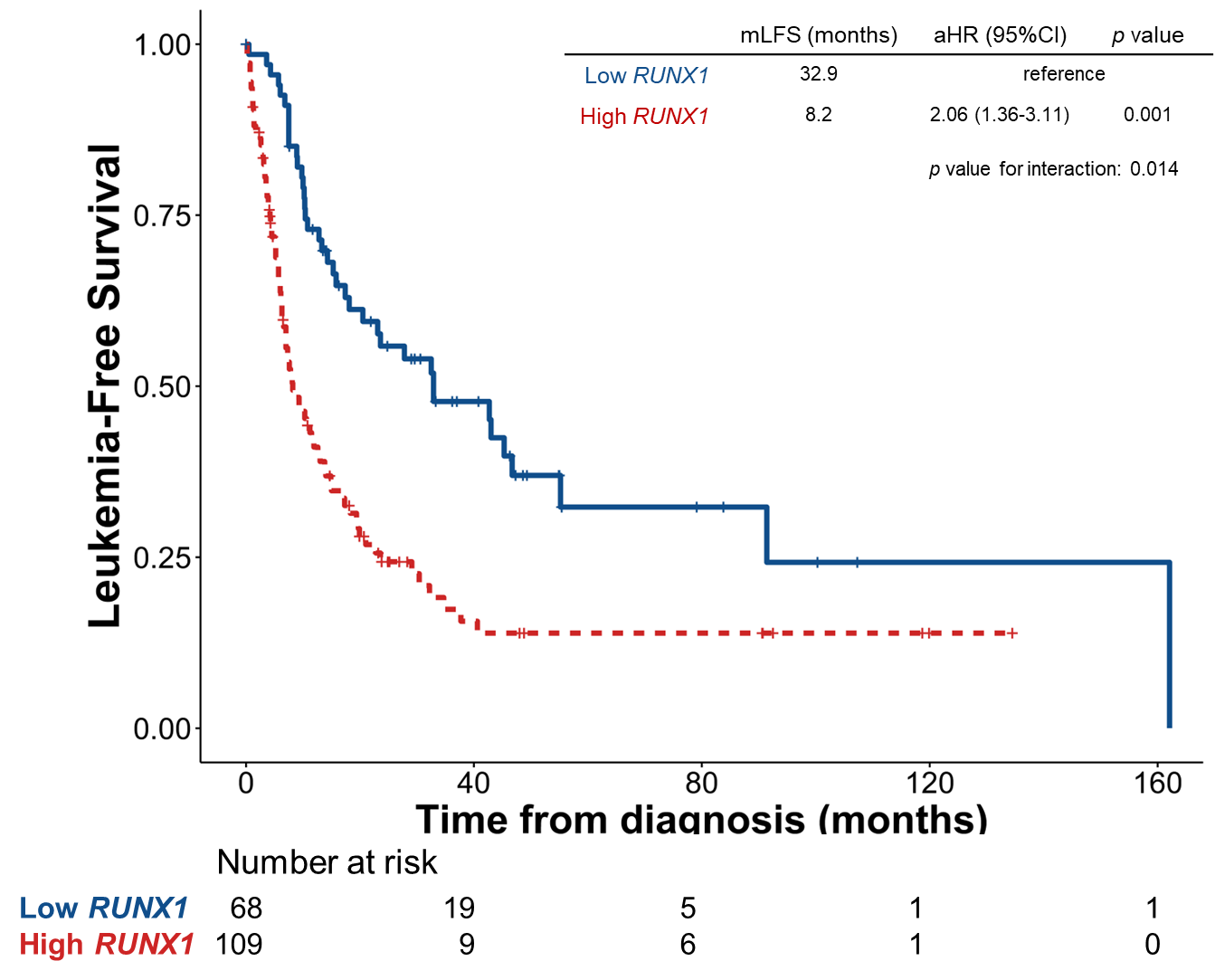** | **d** | **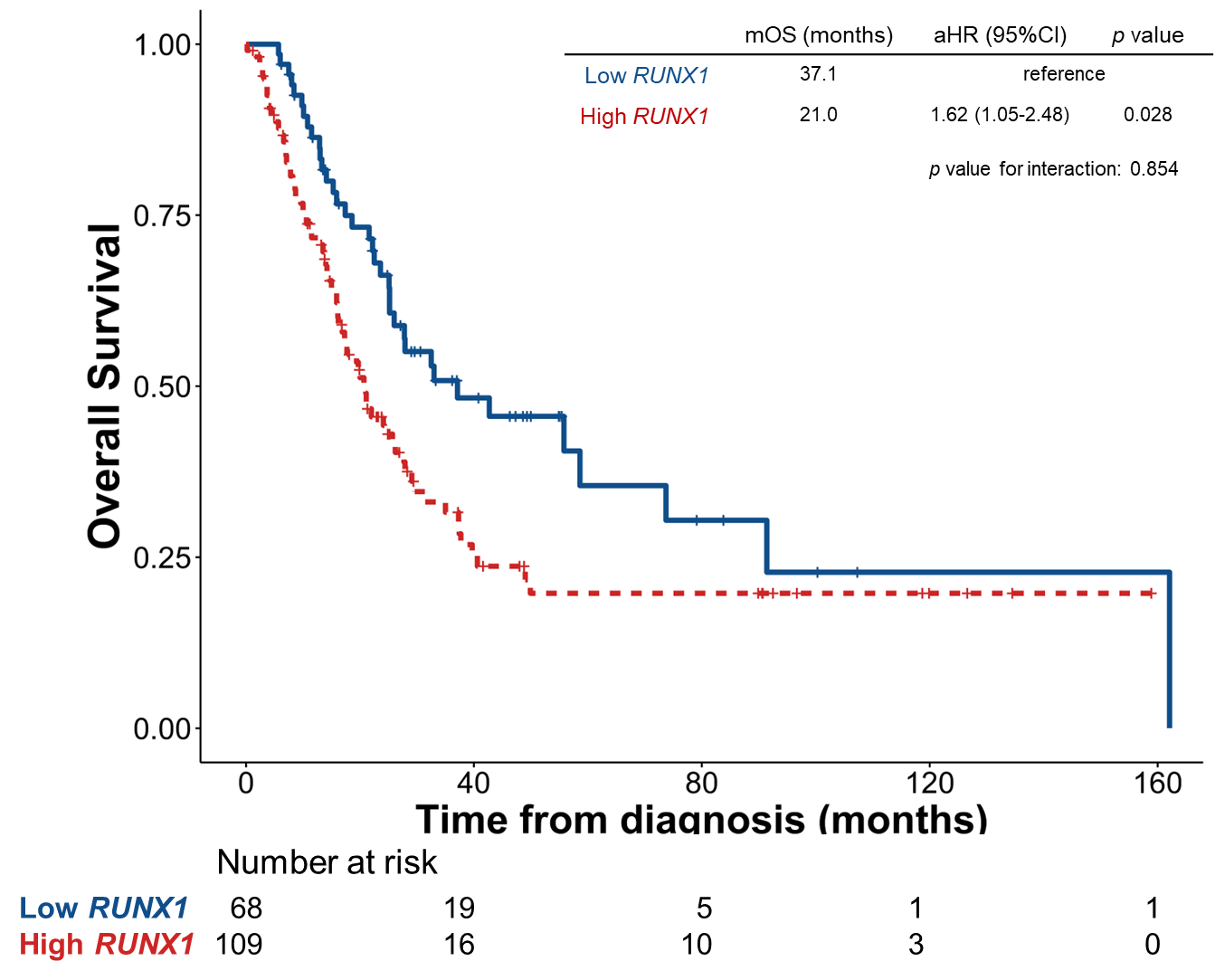** |

**Supplemental Figure 5. Kaplan-Meier survival curves stratified by *RUNX1* expression in the patients receiving hypomethylation agents (HMA).**

(a) LFS and (b) OS of the 134 patients who received HMA. Patients with higher *RUNX1* expression had worse clinical outcomes than those with lower expression.

| **a** | **b** |
| --- | --- |
| **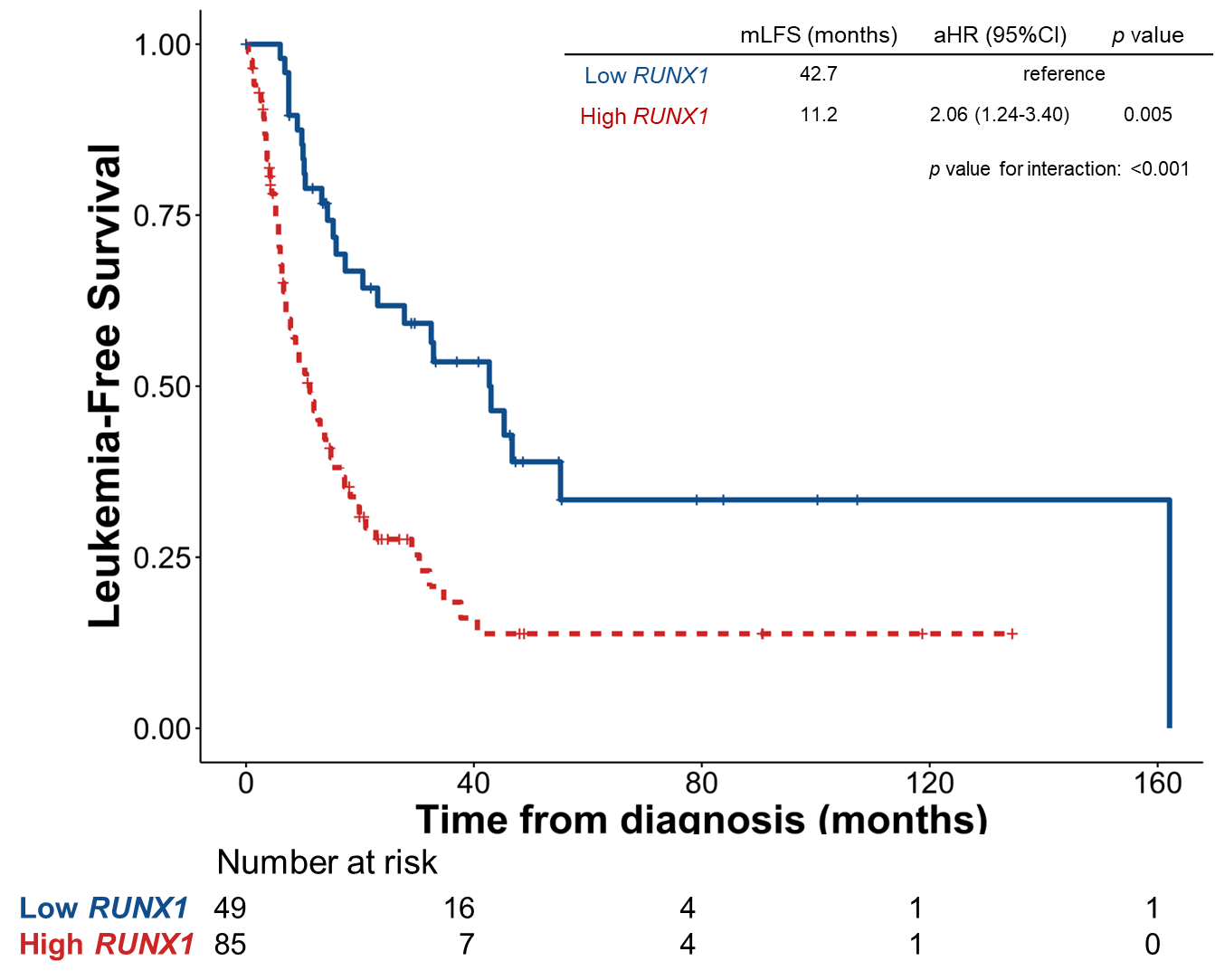** | **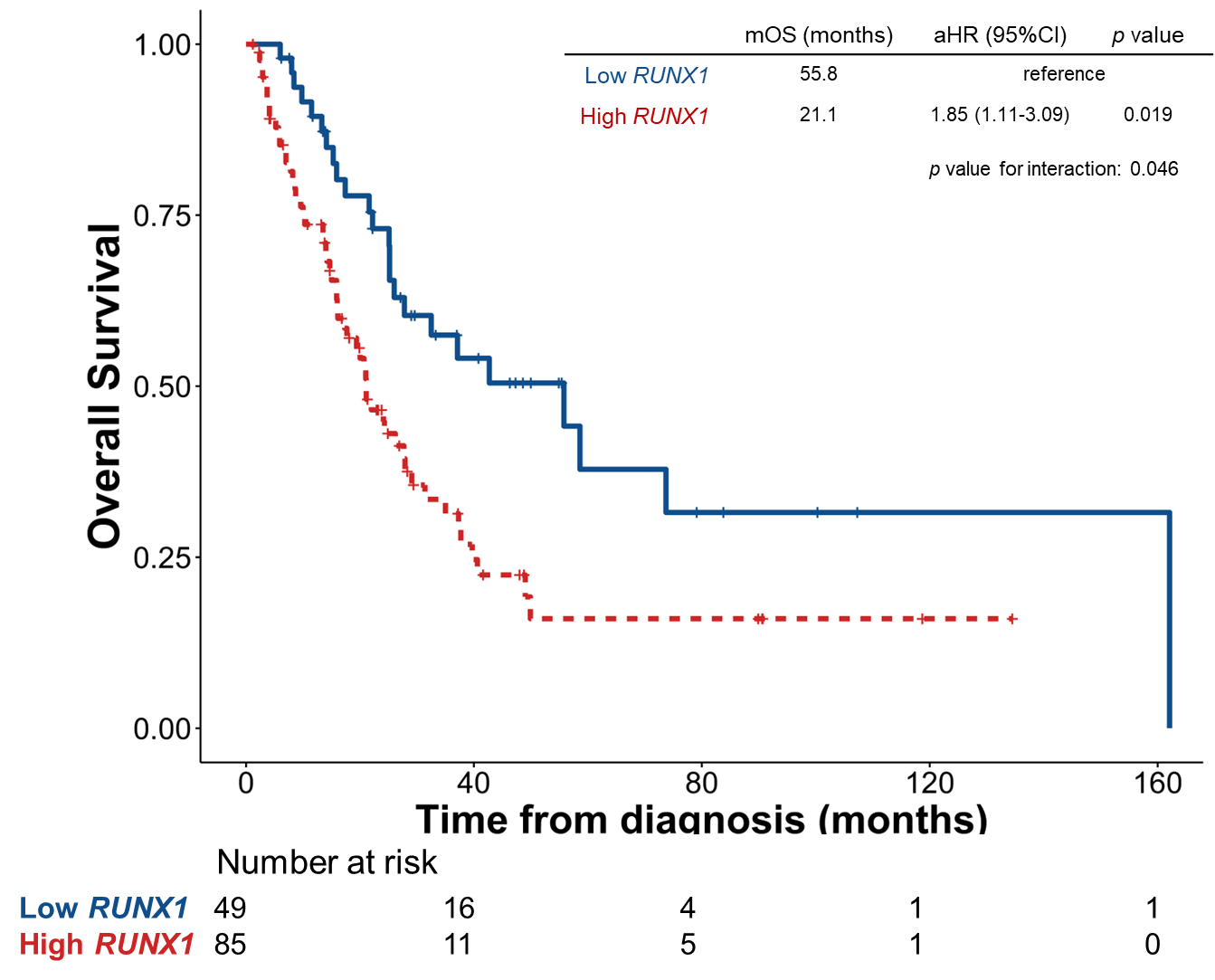** |

**Supplemental Figure 6. Kaplan-Meier plots stratified by *RUNX1* expression and receiving hematopoietic stem cell transplant (HSCT) or not.** (a) LFS and (b) OS of the 59 patients who received HSCT and 282 patients who did not receive HSCT. Patients with higher *RUNX1* expression had comparable OS to those with lower *RUNX1* expression if they received HSCT, inferring that HSCT might partly overcome the detrimental effect of high *RUNX1* expression. Among patients with higher RUNX1 expression, those who received HSCT had better clinical outcomes than those who did not receive HSCT.

| **a** | **b** |
| --- | --- |
| **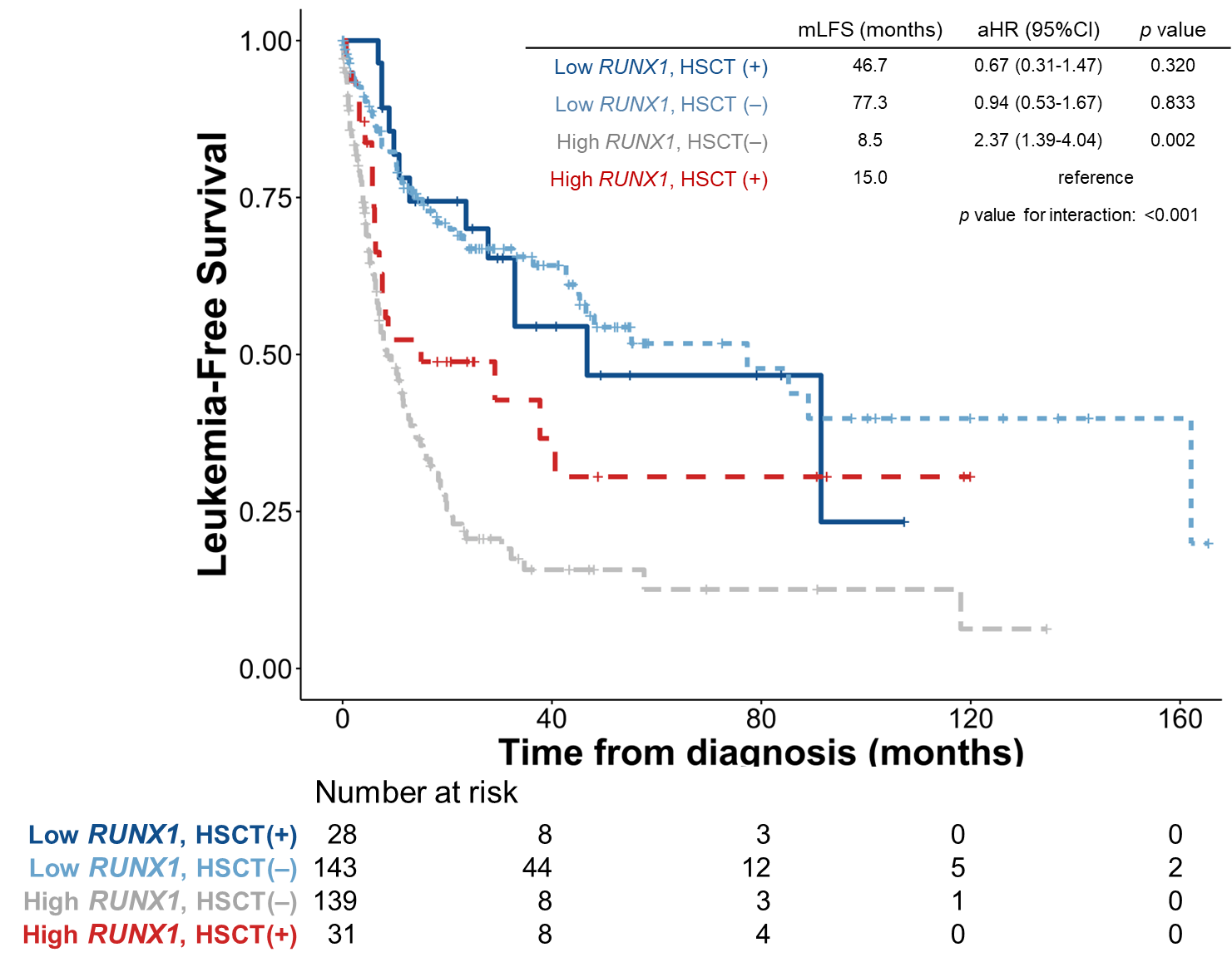** | **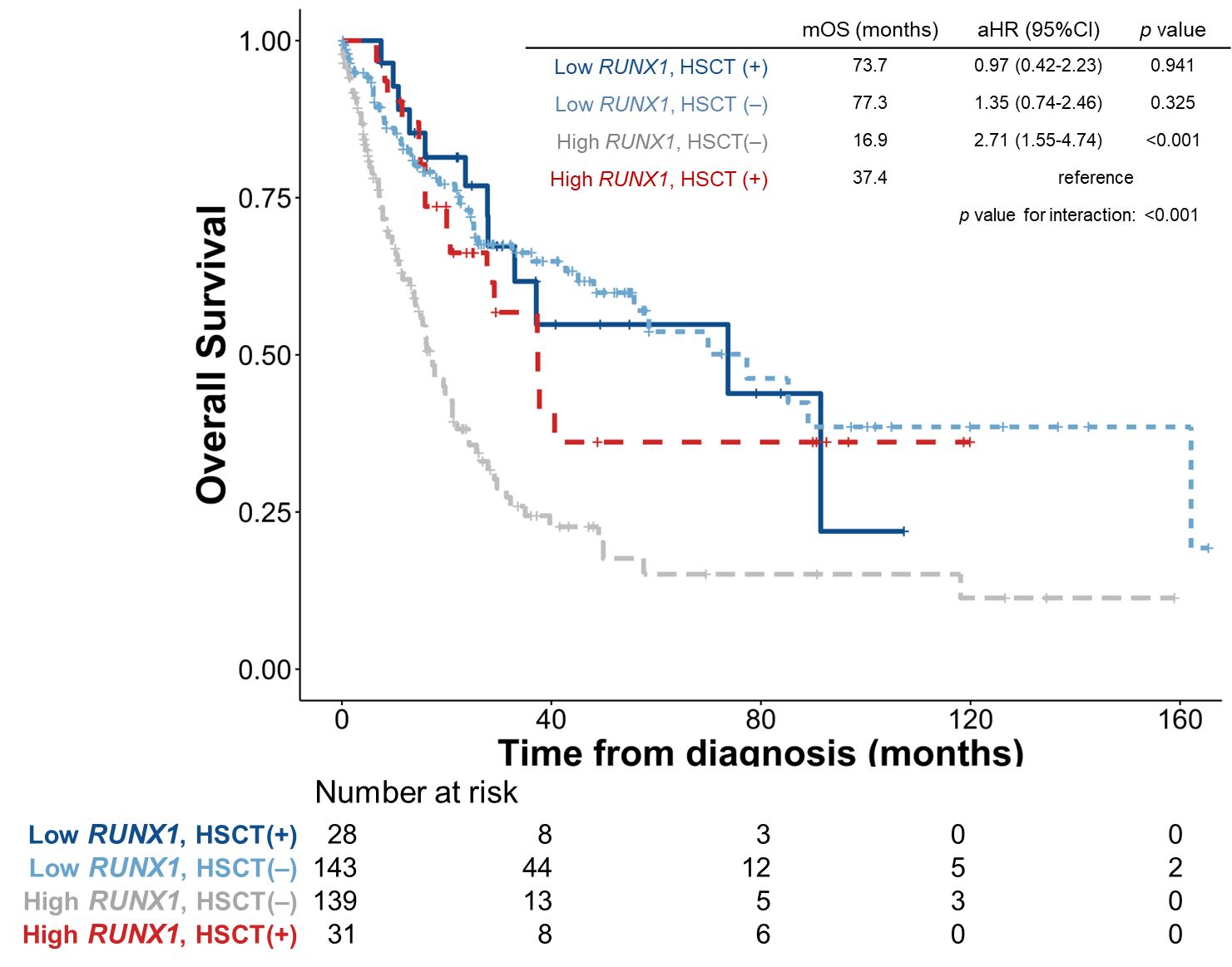** |

**Supplemental Figure 7. Functional enrichment analysis of pathways associated with higher *RUNX1* expression.**

GSEA enrichment plots show a significant association between higher *RUNX1* expression and core enriched hematopoietic stem cells/leukemic stem cells (CE-HSC/LSC) signatures (all *p* values <0.001).

**
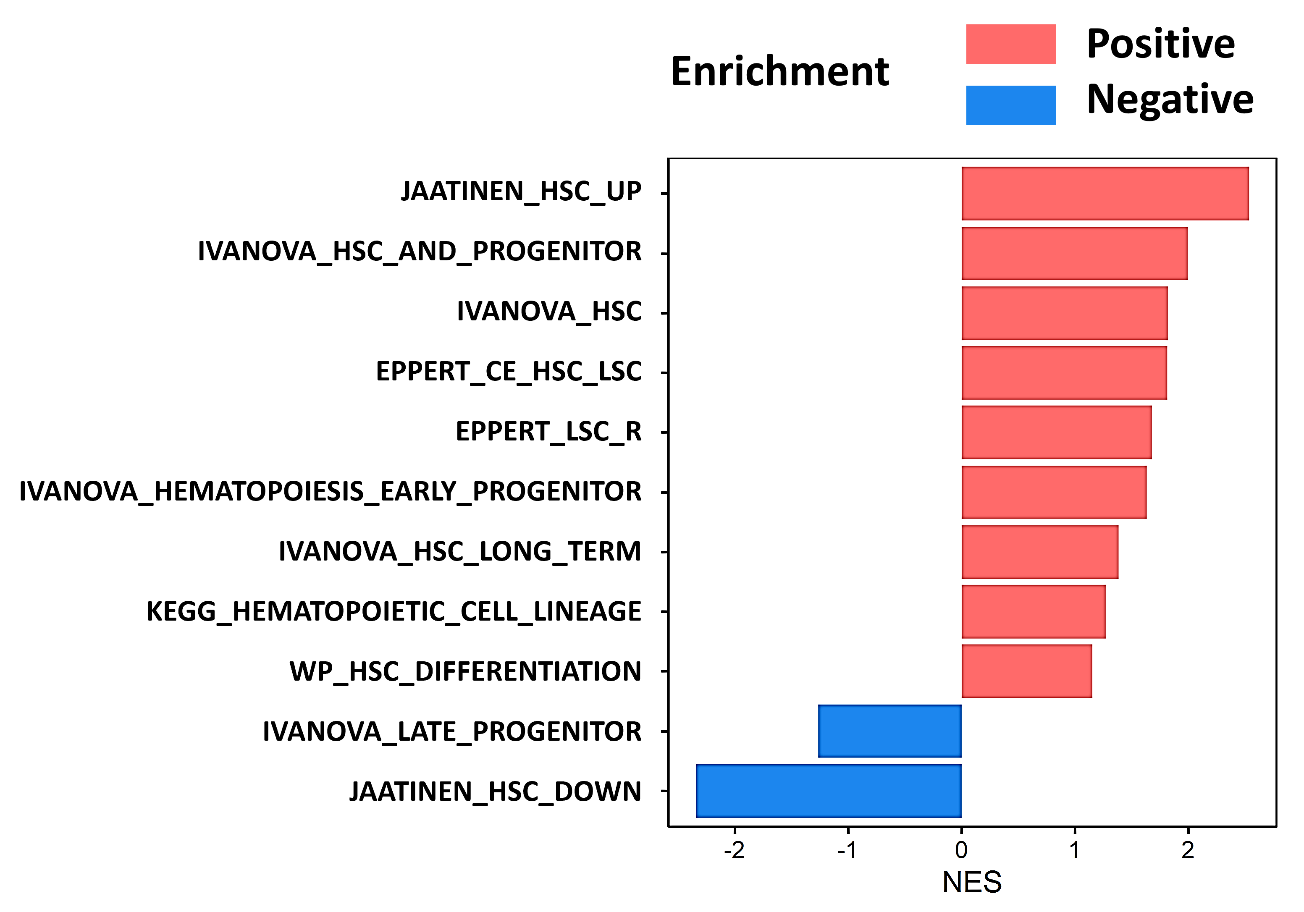

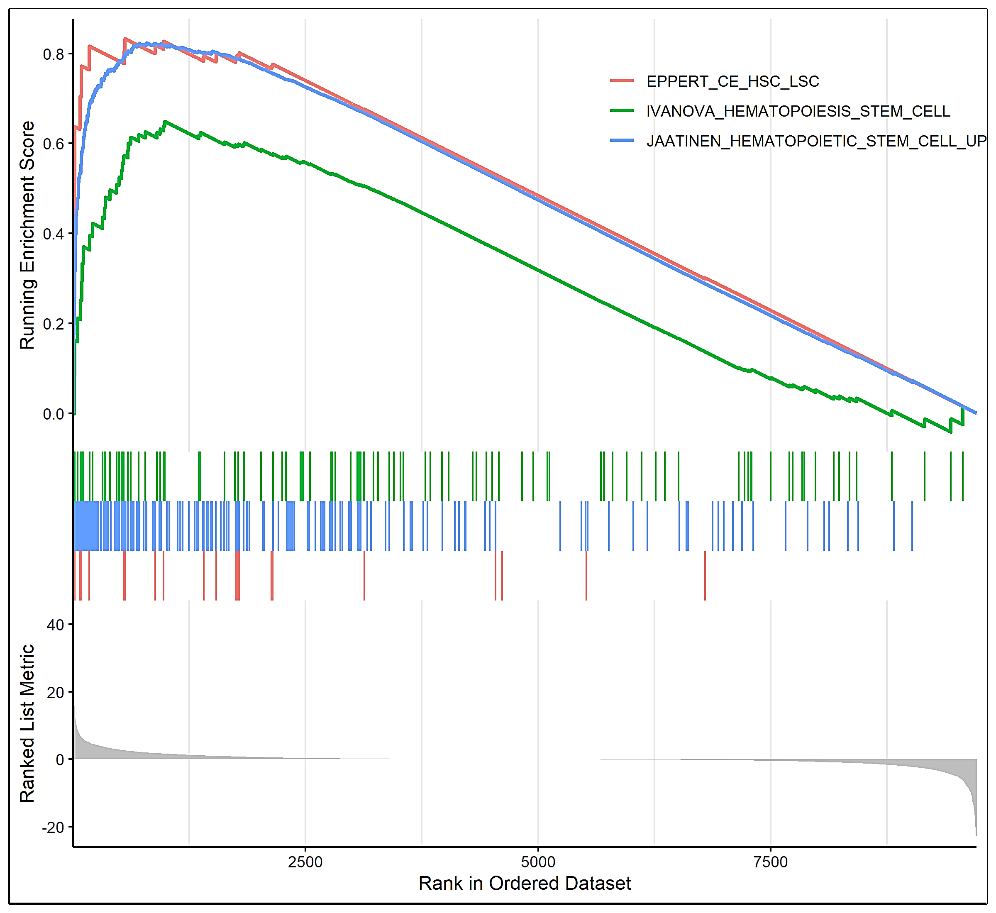
**
